# DNA‐mediated UCP1 overexpression in adipose tissue: A promising anti‐obesity gene therapy

**DOI:** 10.1002/ctm2.70491

**Published:** 2025-10-02

**Authors:** Ze‐Wei Zhao, Longyun Hu, Bigui Song, Qian Wu, Jiejing Lin, Qingqing Liu, Siqi Liu, Jin Li, Molin Wang, Jin Li, Zhonghan Yang

**Affiliations:** ^1^ Department of Biochemistry, School of Medicine Sun Yat‐sen University Shenzhen Guangdong China; ^2^ Department of Geriatrics, The First Affiliated Hospital Sun Yat‐sen University Guangzhou Guangdong China

**Keywords:** adipose tissue, gene therapy, obesity, thermogenesis, UCP1

## Abstract

**Background:**

Obesity has emerged as a global health challenge. Although GLP‐1 receptor agonists are showing considerable promise in weight loss, their clinical utility is partly limited by gastrointestinal adverse reactions and non‐fat weight loss side effects. UCP1‐mediated adipose thermogenesis is a critical process for body temperature maintenance and weight management. However, the lack of effective and specific adipose thermogenesis therapies has restricted its clinical application. We aimed to explore the potential of inducing adipose‐specific UCP1 overexpression via modified plasmids as an innovative therapeutic approach for obesity.

**Methods:**

We replaced the cytomegalovirus (CMV) promoter in the plasmids with two types of adipose‐specific promoters: mouse adiponectin (mADP) promoter and human adiponectin (hADP) promoter, to selectively overexpress UCP1 in adipocytes. The expression level of UCP1, weight loss, metabolic homeostasis and adipose thermogenesis effects were evaluated by immunohistochemistry, western blot, weight measurements, thermography, and comprehensive lab animal monitoring system.

**Results:**

The experiments demonstrated that the mADP promoter‐modified plasmids failed to drive UCP1 overexpression. In contrast, the hADP promoter‐modified *Ucp1* overexpression (hADP‐*Ucp1* OE) plasmids achieved robust adipose‐specific UCP1 protein expression both in vitro and in vivo. In vitro experiments revealed that delivery of the hADP promoter‐modified *UCP1* overexpression (hADP‐*UCP1* OE) plasmids reduced lipid droplet size and enhanced energy consumption in human adipocytes. In obese mice, administration of the hADP‐*Ucp1* OE plasmids resulted in significant weight loss and improved metabolic homeostasis.

**Conclusions:**

These findings highlight the therapeutic potential of hADP‐*UCP1* OE plasmids in obesity management.

**Key points:**

- The hADP promoter‐modified plasmids selectively overexpress protein in adipose tissue.
- Overexpression of UCP1 driven by hADP promoter induces thermogenesis in mouse and human adipocytes in vitro.
- The hADP‐*Ucp1* OE treatment promotes thermogenesis and energy expenditure in mice.
- The hADP‐*Ucp1* OE treatment restrains the development of obesity and glucose intolerance in mice.

## Graphical abstract

This study developed a weight loss gene therapy targeting adipose tissue, which achieved adipose‐specific overexpression of UCP1 by modifying plasmids. This gene therapy strategy achieves adipose‐selective overexpression of UCP1 protein and induces adipose thermogenesis, leading to reduced weight gain, improved metabolic homeostasis, and enhanced energy expenditure in mice.

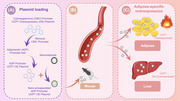

## 1 INTRODUCTION

Based on the findings of the 2024 World Obesity Report, the proportion of overweight or obese adults is anticipated to reach 50% of the global adult population by 2030.^1^ The concerning increase in obesity rates is not limited to wealthy nations; countries with low and middle incomes are also experiencing a rise, frequently alongside undernutrition. Due to the recognised health risks and substantial rise in cases, obesity is a significant global health challenge.^2^, ^3^ The health impacts of obesity are profound and multifaceted. Excessive body weight is a key contributor to the risk of several chronic health issues, including cardiovascular ailments, specific cancers, and breathing disorders.^4^, ^5^, ^6^, ^7^ Obesity is linked to a considerable economic strain, accounting for a significant proportion of healthcare expenditures in many countries.^8^, ^9^, ^10^ Moreover, obesity can have detrimental effects on mental health, standard of living and lifespan.^1^, ^9^, ^11^

The management of obesity is multifaceted, encompassing lifestyle modifications, pharmacological interventions, and surgical procedures. However, lifestyle interventions often yield modest and transient weight loss, with many individuals struggling to maintain long‐term adherence to lifestyle changes.^12^ Pharmacological treatments for obesity have expanded recently, with glucagon‐like peptide‐1 (GLP‐1) receptor agonists (GLP‐1RA) standing out as a promising alternative.^3^, ^13^, ^14^, ^15^, ^16^ These drugs mimic the effects of GLP‐1, a hormone involved in regulating glucose metabolism and appetite. Although GLP‐1RA have shown considerable benefits in weight loss, their clinical utility is partly limited by gastrointestinal side effects such as stomach upset, emesis and diarrhea.^17^, ^18^, ^19^, ^20^ Additionally, these agents may induce non‐fat weight loss side effects, which can be a concern for some patients.^21^, ^22^

Surgical interventions, such as bariatric surgery, are highly effective for obtaining considerable and enduring weight loss in severely obese individuals.^11^, ^23^, ^24^ Nonetheless, these invasive procedures carry inherent risks and are not suitable for all patients.^25^, ^26^, ^27^ The limitations of existing surgical and pharmacological therapies underscore the pressing demand for novel, targeted and efficient weight loss strategies. Weight loss strategies can be generally divided into two major strategies: reducing food intake and promoting energy expenditure.^28^, ^29^ Pharmacological interventions targeting GLP‐1 receptors primarily function to suppress appetite and reduce food intake.^30^, ^31^ Therefore, identifying therapeutic strategies that enhance energy expenditure may provide valuable insights for developing innovative obesity therapies.

A viable approach to enhance energy consumption and combat obesity is to increase the expression of UCP1, thereby promoting adipose thermogenesis.^32^, ^33^, ^34^, ^35^, ^36^ UCP1‐mediated adipose tissue thermogenesis is pivotal for weight management and regulating body temperature.^37^, ^38^, ^39^, ^40^, ^41^, ^42^ However, the clinical implementation of therapies based on adipose thermogenesis has been limited by the lack of effective and specific delivery systems.^32^, ^43^, ^44^, ^45^ In recent years, plasmid therapy has been increasingly validated in terms of feasibility and safety in clinical trials.^46^, ^47^, ^48^ However, to date, no studies have reported the use of plasmid therapy to achieve tissue‐specific overexpression via plasmid modification. In this study, we investigate the feasibility of inducing adipose‐specific UCP1 overexpression via modified plasmids as a novel therapeutic approach to address obesity. Our findings highlight the potential of selectively enhancing adipose thermogenesis as a clinical strategy for obesity management.

## 2 MATERIALS AND METHODS

### 2.1 Study design

This study aimed to devise a technique for selectively overexpressing UCP1 in adipose tissue. We initiated experiments using male C57BL/6J mice. No sample size calculation was conducted; instead, the sample size was determined according to our laboratory's empirical data and preliminary trials. To further verify the statistical reliability, we supplemented the post hoc power analysis, which revealed that at the significance level of *α* = .05, the existing sample size had a statistical efficacy of over 80% for the main outcome indicators of animal experiments, such as body weight and tissue weight. All experiments were conducted using minimum number of mice required for valid statistical analysis. No mice were excluded from the experiment during the process. 3T3‐L1, NIH/3T3, mouse primary adipocytes, 293T cells, and hADSCs were used to assess the in vitro adipocyte specific expression effects of mouse adiponectin (mADP) promoter and human adiponectin (hADP) promoter‐modified plasmids. The expression level of UCP1, weight‐loss, metabolic homeostasis and adipose thermogenesis effects were evaluated by immunohistochemistry, western blot, weight measurements, thermography, and comprehensive lab animal monitoring system. All analyses were conducted without blinding, which is a limitation of this study. We aimed to minimise potential subjective bias through standardised approaches and objective quantification of key outcomes, but we acknowledge that the lack of blinding may still have subtle impacts on the interpretation of results.

### 2.2 Mice

Wild‐type (WT) C57BL/6J mice were acquired from the Laboratory Animal Center of Sun Yat‐sen University. UCP1 knockout (*Ucp1* ^−/−^) mice were established in our previous research.^33^, ^49^ All mice were housed under a 12‐h light‐dark cycle, with the environmental temperature controlled at 21 ± 1°C. Except for those on the HFD, all experimental procedures used 8‐week‐old male mice. To induce obesity, 8‐week‐old male C57BL/6J mice were fed either a normal chow diet (NCD) or a high‐fat diet (HFD, 60% kcal) for 12 weeks.

In vivo experiments were carried out to validate selective overexpression of Ucp1‐6×HIS or luciferase. Mice were administered intraperitoneally (i.p.) with CMV‐*Ucp1*‐6×HIS, mADP‐*Ucp1*‐6×HIS, hADP‐*Ucp1*‐6×HIS, hADP‐*Luc*, or their corresponding control plasmids, all encapsulated with nanomaterials. Injections were given once daily for three consecutive days to validate the adipose‐specific overexpression effect of the mADP‐ and hADP‐modified plasmids. Each injection contained 30 µg plasmid and 48 µL nanomaterial, with the final volume adjusted to 200 µL using PBS.

In vivo experiments were conducted to validate selective overexpression of *Ucp1*. Mice were administered intraperitoneally (i.p.) with either the pcDNA3.1(+)‐hADP promoter‐*Ucp1* overexpression plasmid (hADP‐*Ucp1* OE) or the pcDNA3.1(+)‐hADP promoter‐MCS control plasmid (hADP‐CON), both encapsulated with nanomaterials. The dosing frequency was twice weekly for 2 weeks in NCD‐fed mice, and twice weekly for 12 weeks in HFD‐fed mice, to evaluate the effect of hADP‐*Ucp1* OE on thermogenesis and weight loss. Each injection contained 30 µg plasmid and 48 µL nanomaterial, with the final volume adjusted to 200 µL using PBS.

Mice were fasted for 6 h before conducting intraperitoneal glucose or insulin resistance tests. Intraperitoneal injections of glucose (1 g/kg) or insulin (1U/kg) were administered i.p. at the 0‐min time points. Following administration, blood glucose levels were measured at designated time intervals with a glucometer (OneTouch Ultra, Johnson). In all in vivo studies, each genotype or treatment group used for molecular marker detection (e.g., western blot) should include at least three mice, while those used for functional detection (e.g., body weight measurements) should include at least five mice. All in vivo experiments were independently replicated 2–3 times.

### 2.3 Cell culture

The 3T3‐L1, NIH/3T3, and 293T cell lines were obtained from the Cell Bank of the Chinese Academy of Sciences. All cell lines were authenticated via STR profiling and tested negative for mycoplasma contamination. The Stromal Vascular Fraction (SVF) was isolated from the adipose tissue of wild‐type male C57BL/6J mice. Human adipose‐derived mesenchymal stem cells (hADSCs) were acquired from the National Stem Cell Translational Resource Center. NIH/3T3 and 293T cells were culture in high‐glucose DMEM supplemented with 10% FBS. The culture and induction of adipogenic differentiation of 3T3‐L1, SVF, and hADSCs were conducted according to our previous research.^33^, ^34^ More than 90% of the cells showed obvious lipid droplets under the microscope, indicating differentiation into mature adipocytes. Various cells were transfected with plasmids in vitro using Lipo8000 (C0533, Beyotime).

### 2.4 Construction of plasmid

The sequences information of mADP promoter^50^ (#192360, addgene) and hADP promoter^51^ (#176215, addgene) were obtained from addgene. Furthermore, the corresponding sequences were synthesised via whole‐gene synthesis, and various plasmids were constructed using homologous recombination cloning techniques. Notably, in this study, *Ucp1* specifically refers to the mouse *Ucp1* gene, while *UCP1* specifically refers to the human *UCP1* gene.

### 2.5 Luciferase activity assay

The different treated cells were washed with PBS before detection. Then the different treated cells were incubated with 150 µg/ml D‐Luciferin potassium salt working solution (ST196) for 5 min at 37°C, then were measured using a multimode microplate reader.

### 2.6 Metabolic cages

Oxygen consumption and energy expenditure of mice were assessed using Comprehensive Lab Animal Monitoring System (CLAMS, Columbus). Prior to testing, mice were acclimated to the system for 20–24 h, after which oxygen consumption and energy expenditure were measured continuously over the next 24 h. Mice were singly housed and maintained at 24°C under a 12 h light/dark cycle, with unrestricted access to food and water.

### 2.7 Temperature measurements

The body temperature of mouse was measured at 9:00 am using a rectal probe (LAT‐212, Beijing Lab Anim) connected to a digital thermometer (TH212, Beijing Lab Anim). Allow mice to adapt to the environment before measurement, and then measure body temperature while restraining the mice.

### 2.8 Histology and immunohistochemistry

Liver, iWAT, eWAT and BAT were fixed a universal tissue fixative (G1101, Servicebio). After fixation, the tissues were embedded in paraffin and sliced. For haematoxylin and eosin (HE) staining, the HE staining kit (G1076, Servicebio) was used. For immunohistochemical staining, the antigen repaired paraffin sections were first sealed with goat serum at room temperature for 60 min. Then the blocked sections were incubated overnight with anti‐UCP1 primary antibody (1:2000; 83870‐1‐RR, Proteintech) at 4°C. Subsequently, the EnVision detection system (K500711‐2, DAKO) was used for detection and haematoxylin (G1004, Servicebio) was used for counterstain.

### 2.9 Western blot

Proteins were lysed, extracted and measured using RIPA buffer and BCA kit (BL521, Biosharp). The protein was transferred onto a PVDF membrane (IPVH00010, Millipore) and subsequently incubated with primary antibodies for β‐tubulin (GB15140‐100, Servicebio), UCP1 (83870‐1‐RR, Proteintech) and HIS‐tag (84814‐1‐RR, Proteintech) overnight at 4°C. After incubation with the primary antibody, the PVDF membrane with protein was incubated at room temperature in HRP conjugated secondary antibody (G1213/G1214, Servicebio) for 60 min. After incubation, chemiluminescence imaging was performed using chemiluminescence HRP substrate (WBKLS0100, Millipore) on chemiluminescence imaging system (ChemiDoc MP, Bio‐Rad). After imaging, ImageJ software was used for quantitative analysis of the images.^52^ For all Western blot results, each lane corresponds to an independent sample, and all experiments were repeated 2–3 times.

### 2.10 ELISA

ELISA kit (RXJ202485M, Ruixin) was used to detect the mouse serum insulin levels and ELISA kit (RXJW202703M, Ruixin) was used to detect the mouse serum corticosterone levels.

### 2.11 BODIPY staining

Differently treated adipocytes were washed with PBS before staining. Then adipocytes were incubated with 2 µM BODIPY staining solution (GC42959, GLPBIO) for 15 min at 37°C, and washed with PBS after incubation. The stained adipocytes were visualised under a fluorescence microscope (EVOS M5000, ThermoFisher) with GFP filter (Ex470 nm, Em510 nm).

### 2.12 Mito‐Tracker staining

Differently treated adipocytes were stained with 100 nM Mito‐Tracker staining solution (C1035, Beyotime) for 30 min. The stained adipocytes were washed with PBS and visualised under a fluorescence microscope (EVOS M5000, ThermoFisher) with RFP filter (Ex531 nm, Em593 nm).

### 2.13 2‐deoxy‐D‐glucose (2‐NBDG) uptake capacity

Differently treated adipocytes were washed with PBS before staining. Then adipocytes were stained with 100 µM 2‐NBDG staining solution (HY‐116215, MCE) for 1 h at 37°C, and washed with PBS after incubation. The stained adipocytes were visualised under a fluorescence microscope (EVOS M5000, ThermoFisher) with GFP filter (Ex470 nm, Em510 nm).

### 2.14 Triacylglycerol (TG) detection

Triacylglycerol levels were assayed using a Triglyceride Assay Kit (A110‐1‐1, Nanjing Jiancheng). The triglyceride levels of cells and tissues will be normalised using total protein, and those of cells will be further normalised based on the average triglyceride level of the control group.

### 2.15 Oxygen consumption rate (OCR)

Cells were cultured and induced into mature adipocytes on Seahorse XF24 Microplates. After inducing differentiation of cells into mature adipocytes, transfection was performed, and relevant tests were conducted 36 h after transfection. The OCR was determined using Seahorse XFe‐24 metabolic analyser and mitochondrial stress test kit (103015‐100). The measured data is normalised by total cellular protein.

### 2.16 Infrared thermography

BAT temperature was assessed via infrared thermography under room temperature (21 ± 1°C). Allow the mice to acclimate to the environment before imaging, and perform imaging when the mice are not moving. Representative infrared images of awake mice from the same batch were all captured with a thermal imaging camera (FLIR ONE PRO), with measurements taken at a consistent perpendicular distance from the plane where the mice were positioned. For quantifying the temperature of the interscapular region, the average surface temperature within the interscapular BAT area was calculated using FLIR Tools software. Each treatment group has five mice for imaging.

### 2.17 Complete blood count (CBC) and blood biochemical tests of mice

The CBC and blood biochemical tests of mice were conducted by Wuhan Servicebio Technology Co., Ltd.

### 2.18 Nanomaterials

The nanomaterials used in this study specifically denote Lipo8000 reagent (C0533, Beyotime), which is a type of nanomaterial that can achieve efficient in vitro/in vivo transfection. This nanomaterial loads and protects nucleic acids through cation modification, achieving stable delivery in vivo and entering cells through endocytosis, ultimately releasing nucleic acids inside the cells.

### 2.19 Body composition analysis

The lean and fat masses of mouse were measured via quantitative magnetic resonance (MR) using Echo‐MRITM‐4in1 Body Composition Analyzer (EchoMRI).

### 2.20 Mouse cold challenge

Cold challenge experiments were conducted in climate‐controlled cold chambers. Mice with different treatment were housed in pre‐cooled cages individually without bedding at 4°C, with unrestricted access to pre‐cooled food and water. Body temperatures were measured every 2 h.

### 2.21 Transmission electron microscopy

Sections of brown adipose tissue (BAT) were fixed with electron microscopy fixative (G1102, Servicebio). The subsequent embedding, slicing, and photography were conducted by Wuhan Servicebio Technology Co., Ltd.

### 2.22 Quantitative polymerase chain reaction (qPCR)

Total RNA was isolated by RNA extraction solution (G3013, Servicebio). Reverse transcription of total RNA was conducted with a kit (BL696A, Biosharp). The qPCR analysis using a quantitative PCR kit (BL697A, Biosharp) was performed with the QuantStudio 5 RT PCR system. The primers employed for real‐time PCR are detailed in Table 1.

**TABLE 1:** Primer sequences applied in qPCR.

Gene	Forward primer	Reverse primer
*Ppargc1α*	TATGGAGTGACATAGAGTGTGCT	CCACTTCAATCCACCCAGAAAG
*Prdm16*	TGACCATACCCGGAGGCATA	GGTACCCTGGCTTTGGACTC
*Il6*	TAGTCCTTCCTACCCCAATTTCC	TTGGTCCTTAGCCACTCCTTC
*Tnfα*	CCCTCACACTCAGATCATCTTCT	GCTACGACGTGGGCTACAG
*Glut4*	GTGACTGGAACACTGGTCCTA	CCAGCCACGTTGCATTGTAG
*Gck*	TGAGCCGGATGCAGAAGGA	GCAACATCTTTACACTGGCCT
*Cpt1b*	GCACACCAGGCAGTAGCTTT	CAGGAGTTGATTCCAGACAGGTA
*β‐Actin*	TGTCCACCTTCCAGCAGATGT	AGCTCAGTAACAGTCCGCCTAGA

### 2.23 Single‐cell nuclear RNA sequencing (snRNA‐seq) data analysis

The snRNA‐seq data analysis was carried out following a previous study.^53^

### 2.24 Statistical analysis

All data are expressed as mean ± *SEM*. The student's *t*‐test was utilised for comparisons between two groups. For comparisons involving more than two groups, one‐way analysis of variance (ANOVA) or two‐way ANOVA was conducted using GraphPad Prism 9.0 software. Results from metabolic cage experiments were analysed via analysis of covariance (ANCOVA). For each parameter in the presented data, statistical significance is denoted as follows: NS (no significance), *p  <  .05, **p <  .01, ***p <  .001 and ****p <  .0001. *p* <  .05 is considered significant.

## 3 RESULTS

### 3.1 The hADP promoter‐modified plasmid achieves selective overexpression of UCP1 protein in mouse adipocytes

**FIGURE 1:**
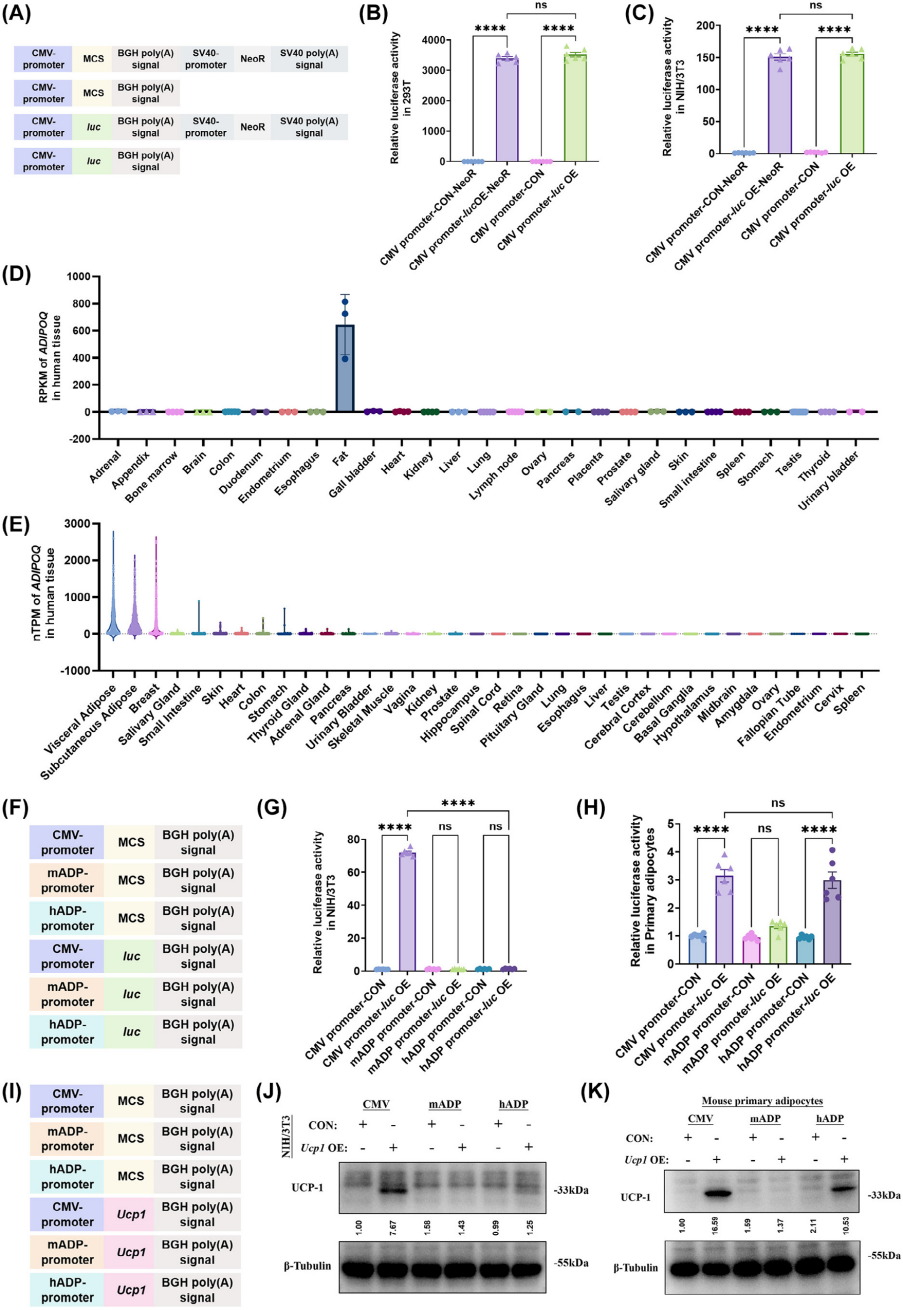
The hADP promoter‐modified plasmid achieves selective overexpression of target protein in mouse adipocytes, in contrast to the CMV promoter. (A, F, I) Schematic diagram of gene elements of different plasmids. (B) Relative luciferase activity analysis in 293T cells transfected with different plasmids. (C, G) Relative luciferase activity analysis in NIH/3T3 cells transfected with different plasmids. (D) The RPKM of *ADIPOQ* gene in human tissues from HPA RNA‐seq normal tissues (PRJEB4337). (E) The nTPM of *ADIPOQ* gene in human tissues from GTEx Portal database. (H) Relative luciferase activity analysis in mouse primary adipocytes transfected with different plasmids. (J) Western blot analysis for UCP1 protein level in NIH/3T3 cells transfected with different plasmids. The ImageJ software was used for grey scanning. (K) Western blot analysis for UCP1 protein level in mouse primary adipocytes transfected with different plasmids. The ImageJ software was used for grey scanning. UCP1: uncoupling protein 1; luc: luciferase; CON: control; OE: overexpression; SV40: simian virus 40; NeoR: neomycin resistance gene; CMV: cytomegalovirus; mADP: mouse adiponectin; hADP: human adiponectin; NS: no significance; ANOVA: one‐way analysis of variance. All data are presented as mean ± *SEM*. Statistical significance was determined by one‐way ANOVA (B‐C and G‐H).

To construct an appropriate modified plasmid, the widely used high‐copy plasmid pcDNA3.1(+) was chosen as a template for modification. The plasmid pcDNA3.1 contains two eukaryotic promoters: the CMV promoter, which drives the expression of the target protein, and the simian virus 40 (SV40) promoter, which drives the expression of neomycin resistance gene (NeoR). To eliminate potential interference of the SV40 promoter with subsequent specific overexpression mediated by the modified plasmid, the sequences related to NeoR ranging from the SV40 promoter to the SV40 polyadenylation (ploy(A)) signal were removed from the plasmid pcDNA3.1(+) (Figures 1A and S1A). The results of western blot analysis and luciferase activity indicated that the excision of the NeoR‐related sequences had no impact on the overexpression efficiency of the plasmid (Figures 1B and C and S1B and C). Therefore, subsequent plasmid modifications were carried out on the pcDNA3.1(+) plasmid without the NeoR‐related sequences.

The results of *ADIPOQ* gene obtained from HPA RNA‐seq normal tissues (PRJEB4337, Figure 1D) and GTEx Portal database (Figure 1E) showed that adiponectin was specifically expressed in adipose tissue within various human tissues, which implies that plasmid modified with adiponectin promoter might be capable of driving adipose‐specific overexpression. To achieve adipose‐specific overexpression, we replaced the CMV promoter sequence with mADP promoter and hADP promoter sequences, respectively. Unexpectedly, the results of the luciferase activity assay (Figure 1F–H) and western blot analysis (Figure 1I–K) showed that replacing the CMV promoter sequence of the plasmids with the mADP promoter sequence did not drive overexpression of the target protein in NIH/3T3 cells and pre‐adipocytes, nor did it drive overexpression of the target protein in mouse adipocytes (Figures 1F–K and S1D and E). This may be due to the lack of long‐range enhancer elements at endogenous genomic sites in the exogenous mADP promoter obtained from addgene, which is crucial for maintaining high transcriptional activity and leads to weak driving of UCP1 expression by mADP promoter. Fortunately, the exogenous hADP promoter obtained from Addgene contains corresponding endogenous enhancer elements. Interestingly, the plasmids with hADP promoter replacement could overexpress the target protein specifically in mouse adipocytes, but not in mouse non‐adipocytes such as NIH/3T3 cells and pre‐adipocytes (Figures 1F–K and S1D–H). The presence or absence of enhancer elements may be the main reason why mADP promoter cannot drive the overexpression of target genes in adipocytes, while the hADP promoter can.

### 3.2 The hADP promoter‐modified plasmid achieves selective overexpression of UCP1‐6×HIS protein in mouse adipose

**FIGURE 2:**
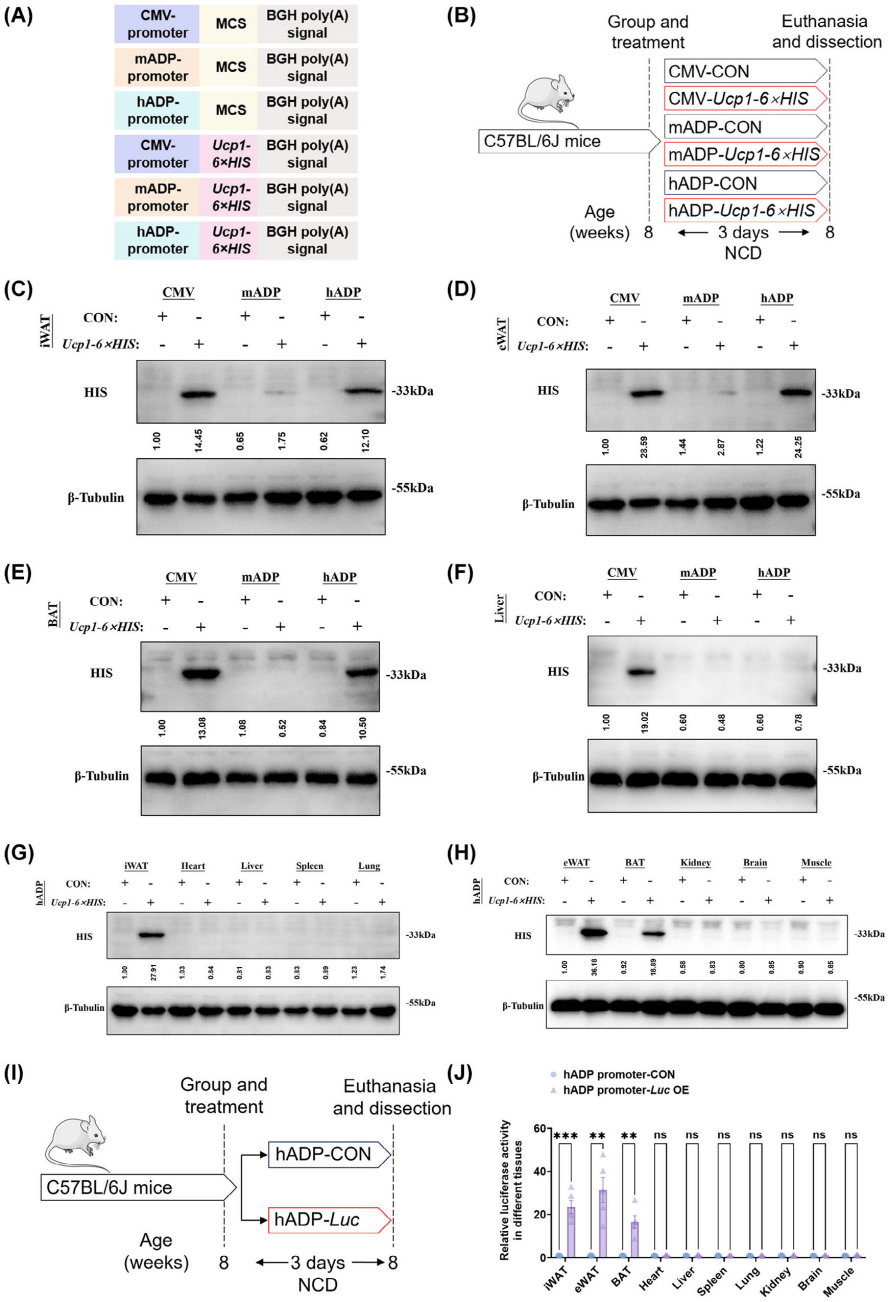
The hADP promoter‐modified plasmid achieves selective overexpression of UCP1‐6×HIS protein in mouse adipose tissue, in contrast to the CMV promoter. (A) Schematic diagram of gene elements of different plasmids. (B) Experimental schematic. C57BL/6J mice fed with a NCD for eight weeks were injected with CMV‐*Ucp1‐6×HIS* (pcDNA3.1(+)‐CMV promoter‐*Ucp1*‐*6×HIS* plasmids encapsulated with nanomaterials), mADP‐*Ucp1‐6×HIS* (pcDNA3.1(+)‐mADP promoter‐*Ucp1‐6×HIS* plasmids encapsulated with nanomaterials), hADP‐*Ucp1‐6×HIS* (pcDNA3.1(+)‐hADP promoter‐*Ucp1‐6×HIS* plasmids encapsulated with nanomaterials) and corresponding control plasmids encapsulated with nanomaterials i.p. once a day for 3 days. (C–F) Western blot analysis for UCP1‐6×HIS protein level in iWAT (C), eWAT (D), BAT (E) and liver (F) from differently treated mice. The ImageJ software was used for grey scanning. (G, H) Western blot analysis for UCP1‐6×HIS protein level in different tissues from differently treated mice. The ImageJ software was used for grey scanning. (I) Experimental schematic. C57BL/6J mice fed with a NCD for 8 weeks were injected with hADP‐*Luc* (pcDNA3.1(+)‐hADP promoter‐Luc plasmids encapsulated with an in vivo transfection reagent) and corresponding control plasmids encapsulated with an in vivo transfection reagent i.p. once a day for 3 days. (J) Relative luciferase activity analysis in different treated mouse tissues. UCP1: uncoupling protein 1; CON: control; CMV: cytomegalovirus; mADP: mouse adiponectin; hADP: human adiponectin. All data are presented as mean ± *SEM*. Statistical significance was determined by unpaired two‐tailed Student's *t*‐test (J).

To further investigate whether the modified plasmid can achieve adipose‐specific overexpression in vivo, we constructed plasmids with three different promoter sequences, each plasmid carrying the coding DNA sequence (CDS) of *Ucp1‐6×HIS* (Figure 2A). These overexpression plasmids were individually mixed with nanomaterials and intraperitoneally injected into separate groups of mice (Figure 2B). Western blot analysis revealed that the plasmid modified with the mADP promoter exhibited weak overexpression of the UCP1‐6×HIS protein in white adipose tissue (Figure 2C and D). In contrast, the hADP promoter‐modified plasmid demonstrated robust overexpression of the UCP1‐6×HIS protein in inguinal white adipose tissue (iWAT), epididymal white adipose tissue (eWAT), and brown adipose tissue (BAT) (Figure 2C–E). Notably, neither the mADP nor the hADP promoter‐modified plasmids induced detectable expression of the target protein in the liver (Figure 2F). Given the relatively weak overexpression efficiency of the mADP promoter‐modified plasmid in vivo, we selected the hADP promoter‐modified plasmid for further validation. The results of western blot analysis indicated that the hADP promoter‐modified *Ucp1‐6×HIS* overexpression plasmid achieved specific overexpression of the UCP1‐6×HIS protein in adipose tissues, while no detectable expression was observed in non‐adipose tissues (Figure 2G and H). Moreover, the results of luciferase activity assay indicated that the hADP promoter‐modified *Luciferase* (*Luc*) overexpression plasmid achieved specific overexpression of the Luciferase protein in adipose tissues, while no detectable expression was observed in non‐adipose tissues (Figure 2I and J).

### 3.3 Overexpression of UCP1 driven by hADP promoter induces thermogenesis in mouse adipocytes

**FIGURE 3:**
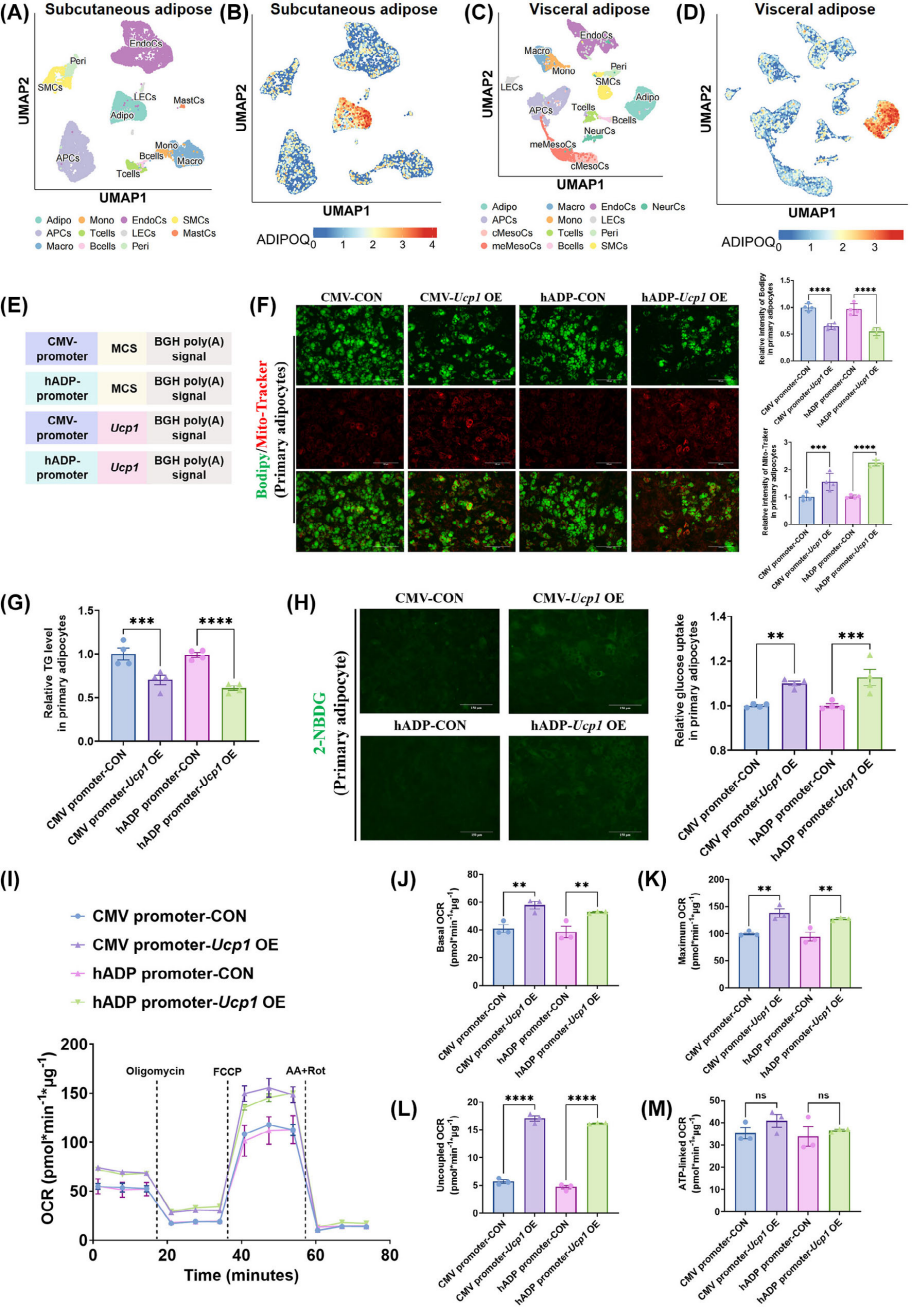
Overexpression of UCP1 driven by the hADP promoter induces thermogenesis in mouse primary adipocytes, consistent with the CMV promoter. (A) Uniform manifold approximation and projection (UMAP) of 19 186 nuclei representing human subcutaneous adipose tissue coloured by broad cell types. (B) Feature plot showing expression of *ADIPOQ* in human subcutaneous adipose tissue. (C) UMAP of 28 931 nuclei representing human visceral adipose tissue coloured by broad cell types. (D) Feature plot showing expression of *ADIPOQ* in human visceral adipose tissue. (E) Schematic diagram of gene elements of different plasmids. (F) BODIPY green staining for lipid droplet and Mito‐Tracker red staining for mitochondria in mouse primary mature adipocytes and staining intensity analysis diagram. Scale bars, 150 µm. (G) The level of intracellular triglyceride in mouse primary mature adipocytes. (H) Glucose uptake assay in mouse primary mature adipocytes and staining intensity analysis diagram. (I–M) OCR (I) and quantification of respiratory profile (J–M) of mouse primary mature adipocytes. UCP1: uncoupling protein 1; CON: control; OE, overexpression; TG: triglyceride; OCR: oxygen consumption rate; 2‐NBDG: 2‐deoxy‐D‐glucose; CMV: cytomegalovirus; hADP: human adiponectin; NS: no significance; ANOVA: one‐way analysis of variance. All data are presented as mean ± *SEM*. Statistical significance was determined by one‐way ANOVA (F–H and J–M).

The results of single‐cell nuclear RNA sequencing (snRNA‐seq) indicated that the cells mainly expressing adiponectin in human subcutaneous adipose tissue (Figure 3A and B) and human visceral adipose tissue (Figure 3C and D) were both adipocytes. Furthermore, we sought to determine whether the hADP promoter‐modified *Ucp1* overexpression (hADP‐*Ucp1* OE) plasmid could induce thermogenesis in mouse adipocytes. To this end, 3T3‐L1 adipocytes and primary mouse adipocytes were transfected with *Ucp1* overexpression plasmids driven by either the CMV promoter or the hADP promoter (Figure 3E). The results of cell fluorescence imaging and intracellular triglyceride detection showed a notably decrease in both the quantity of lipid droplets and intracellular triglyceride levels after *Ucp1* overexpression mediated by the hADP promoter‐modified plasmid, similar to that observed with the CMV promoter‐driven plasmid (Figures 3F and G and S2A and B). Additionally, the glucose uptake ability of adipocytes was significantly enhanced following *Ucp1* overexpression driven by the hADP promoter‐modified plasmid, comparable to the effect of the CMV promoter‐driven plasmid (Figures 3H and S2C). The qPCR analysis results indicated that the expression of *Glut4* and *Cpt1b* increased in primary adipocytes after UCP1 overexpression, while the expression level of Gck showed no significant change (Figure S2D and E).

To further evaluate the impact of *Ucp1* overexpression on cellular energy consumption, the cellular OCR was measured. The results of OCR indicated that treatment with the hADP‐*Ucp1* OE plasmid significantly increased the basal, maximal, and uncoupled OCR in mouse adipocytes (Figure 3I–L), while having no significant effect on ATP‐linked OCR (Figure 3M). In addition, we found that even on primary adipocytes derived from *Ucp1* ^−/−^ mice, the hADP‐Ucp1 OE plasmid can still drive overexpression of UCP1 (Figure S2G). The above results indicate that the hADP‐*Ucp1* OE plasmid can achieve similar effects to the CMV‐*Ucp1* OE plasmid in mouse adipocytes. Moreover, compared to CMV promoters, plasmids modified with the hADP promoter can achieve adipose‐specific overexpression in vivo (Figure 2), representing its key advantage. Collectively, these findings suggest that *Ucp1* overexpression driven by the hADP promoter‐modified plasmid effectively induces thermogenesis and lipid droplet reduction in mouse adipocytes in vitro.

### 3.4 The hADP‐*Ucp1* OE treatment promotes thermogenesis and energy expenditure

**FIGURE 4:**
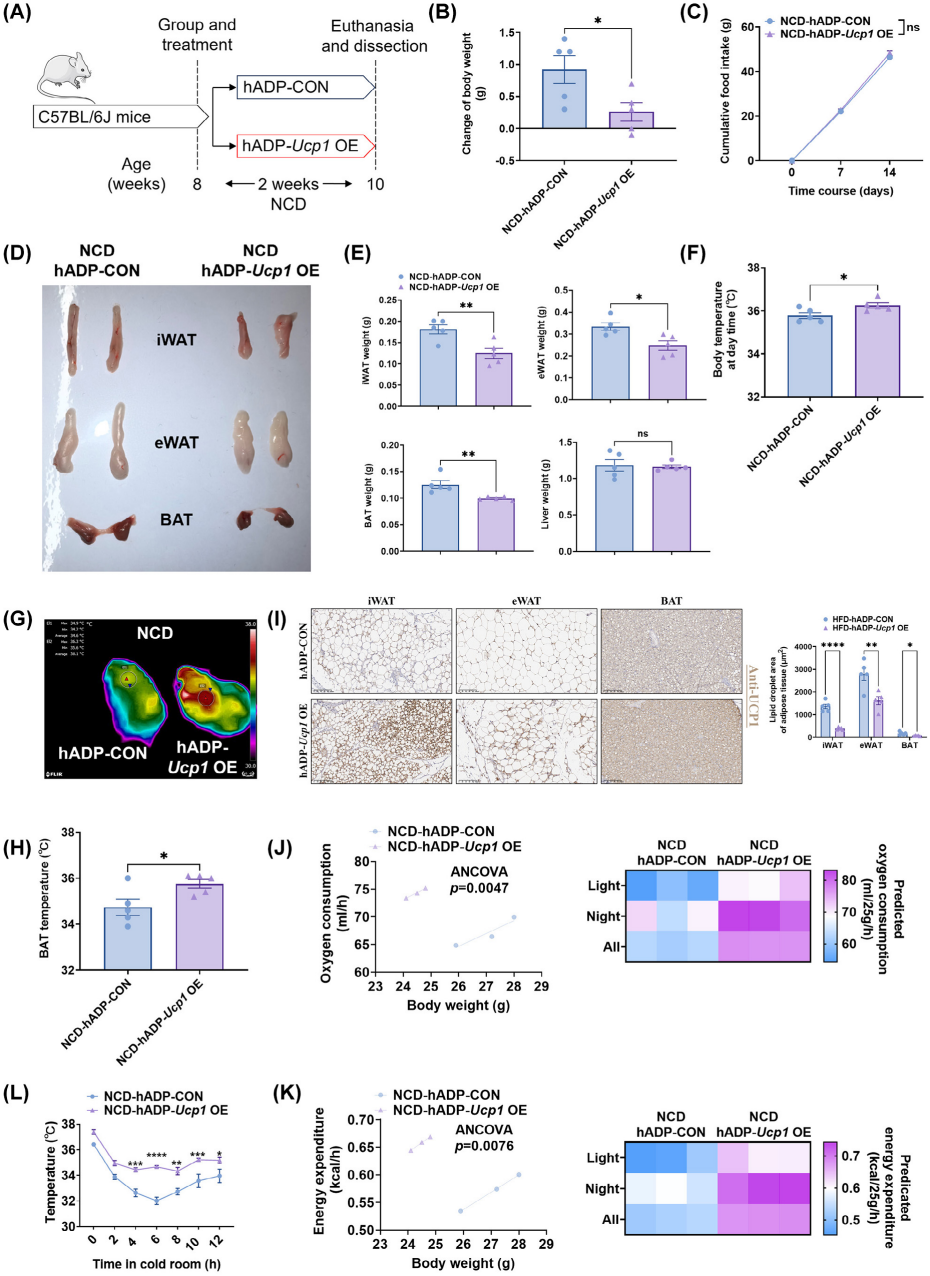
The hADP‐*Ucp1* OE treatment promotes thermogenesis and energy expenditure in mice fed with a NCD. (A) Experimental schematic. C57BL/6J mice fed with a NCD for 8 weeks were injected with hADP‐*Ucp1* OE plasmids encapsulated with nanomaterials and hADP‐CON plasmids encapsulated with nanomaterials i.p. twice a week for 2 weeks (*n* = 5 per treatment). (B) Change of body weight in mice treated differently. (C) Cumulative food intake of mice treated differently. (D) Representative images of iWAT, eWAT and BAT from mice treated differently. (E) The iWAT, eWAT, BAT and liver weight of mice treated differently. (F) Body temperature of mice treated differently. (G, H) Representative thermal image (G) and BAT temperature (H) of mice treated differently. (I) Representative images of iWAT, eWAT and BAT stained with UCP1 (Scale bars, 100 µm). (J, K) The oxygen consumption (J) and the calorie consumption (K) of C57BL/6J mice fed with a NCD for 8 weeks (*n* = 3 per treatment). Data in panels J and K have been analysed using ANCOVA with oxygen consumption (J)/ calorie consumption (K) as dependent variable, group as fixed variable and body mass as covariate. (L) The rectal temperature of mice treated differently. UCP1: uncoupling protein 1; CON: control; OE, overexpression; NCD: normal‐chow diet; iWAT: inguinal white adipose tissue; eWAT: epididymal white adipose tissue; BAT: brown adipose tissue; hADP: human adiponectin; NS: no significance; ANOVA: one‐way analysis of variance. All data are presented as mean ± *SEM*. Statistical significance was determined by unpaired two‐tailed Student's *t*‐test (B, E, F, H) and two‐way ANOVA (C, L). Metabolic cage results were analysed by performing ANCOVA (J, K).

Given the in vivo evidence demonstrating that hADP promoter‐modified overexpression plasmids can selectively and effectively overexpress target proteins in adipose tissue (Figure 2), and the in vitro findings showing that hADP‐*Ucp1* OE plasmid treatment induces thermogenesis and reduces lipid droplets in mouse adipocytes (Figure 3), we selected the hADP‐*Ucp1* OE plasmid for subsequent in vivo experiments in mice fed a normal chow diet (NCD) (Figure 4A). Before the experiment, we tested the time course of protein overexpression driven by hADP‐modified plasmids using the hADP‐*Luc* OE plasmid. Results showed that following injection of the hADP‐*Luc* OE plasmid, overexpressed luciferase protein in iWAT persisted for approximately 3–4 days and was undetectable by day 5 (Figure S2H). Therefore, we selected a dosing frequency of twice per week for subsequent animal experiments.

Following treatment with the hADP‐*Ucp1* OE plasmid, mice exhibited significantly reduced weight gain without changes in food intake (Figure 4B and C). Additionally, the weights of iWAT, eWAT and BAT were significantly decreased in mice treated with the hADP‐*Ucp1* OE plasmid compared to controls (Figure 4D and E). These treated mice also showed elevated body temperature (Figure 4F) and BAT temperature (Figure 4G and H). Moreover, hADP‐*Ucp1* OE plasmid treatment significantly increased UCP1 protein expression in iWAT, eWAT, and BAT (Figures 4I and S3A–F), while reducing adipocyte size in these adipose tissues (Figures 4I and S3G). We further measured oxygen consumption (Figure 4J) and energy expenditure (Figure 4K) and found that hADP‐*Ucp1* OE plasmid treatment significantly enhanced overall energy expenditure in mice. In addition, the thermogenesis of hADP‐*Ucp1* OE plasmid‐treated mice was enhanced as indicated by tolerance to cold challenge (Figure 4L). Collectively, these results demonstrate that hADP‐*Ucp1* OE plasmid treatment effectively induces adipose thermogenesis and increases energy expenditure in mice.

### 3.5 The hADP‐*Ucp1* OE treatment restrains the development of obesity and glucose intolerance

**FIGURE 5:**
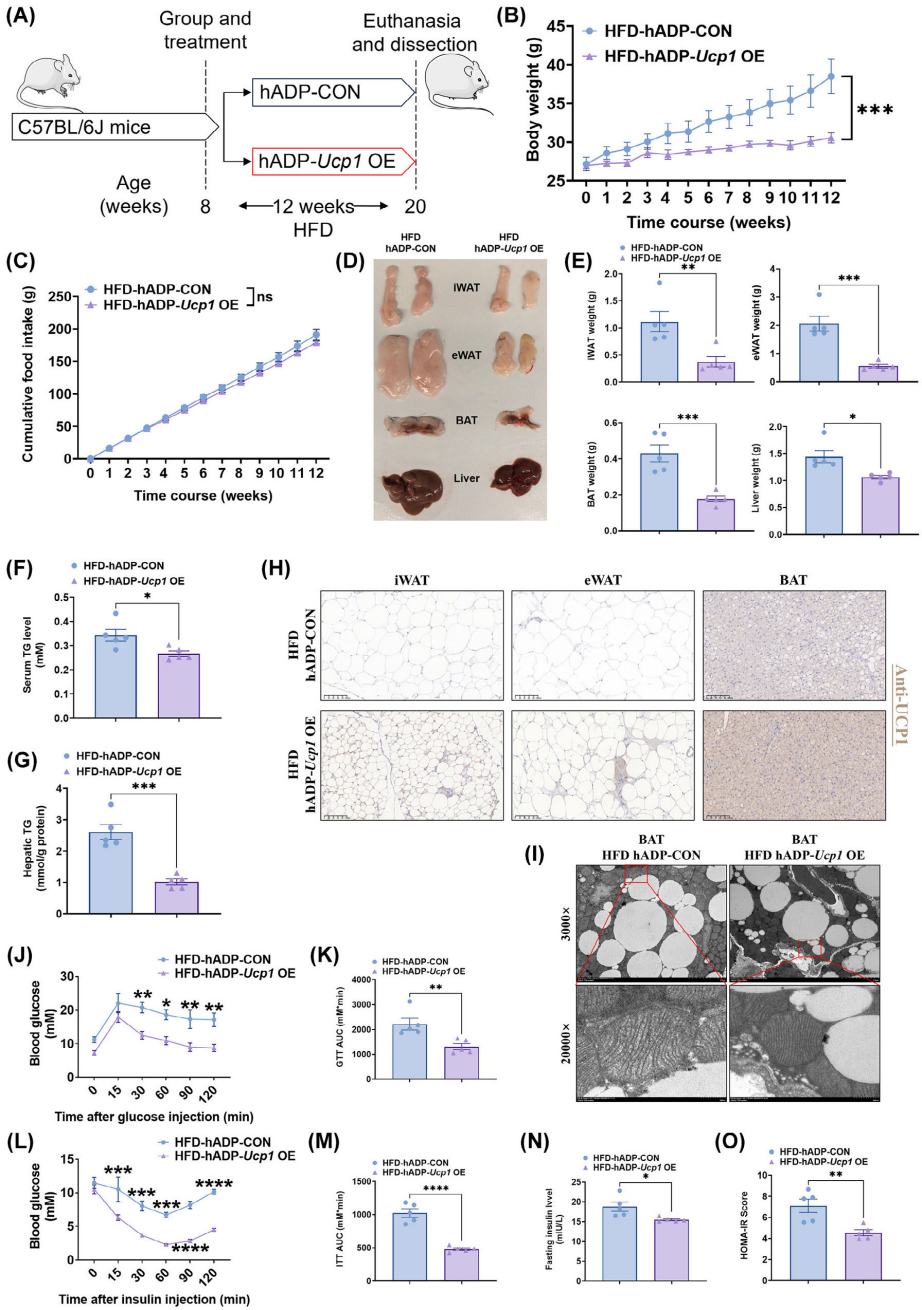
The hADP‐*Ucp1* OE treatment restrains the development of obesity and glucose intolerance in mice fed with a HFD. (A) Experimental schematic. Eight‐week‐old C57BL/6J mice fed with a HFD for 12 weeks were injected with hADP‐*Ucp1* OE plasmids encapsulated with nanomaterials and hADP‐CON plasmids encapsulated with nanomaterials i.p. twice a week for 12 weeks (*n* = 5 per treatment). (B) Change of body weight in mice treated differently. (C) Cumulative food intake of mice treated differently. (D) Representative images of iWAT, eWAT, BAT and liver from mice treated differently. (E) The iWAT, eWAT, BAT and liver weight of mice treated differently. (F, G) The TG level of serum (F) and liver (G) from different treated mice. (H) Representative images of iWAT, eWAT and BAT stained with UCP1 (Scale bars, 100 µm). (I) Representative transmission electron microscope images of BAT. (J) Glucose tolerance test (GTT) assay was conducted by intraperitoneal injection of glucose (1 g/kg) and measurement of blood glucose concentration with a OneTouch Ultra Glucometer at designed time points in 6 h fasted mice. (K) The calculated area under the curve (AUC) of GTT. (L) Insulin tolerance test (ITT) assay was done by intraperitoneal injection of insulin (1U/kg) and measurement of blood glucose concentration by a OneTouch Ultra Glucometer at designed time points in 6 h fasted mice. (M) The calculated AUC of ITT. (N, O) The fasting serum insulin (N) and HOMA‐IR (O) in mice. HOMA‐IR = Fasting glucose level (mmol/L) * Fasting insulin level (mIU/L) /22.5. UCP1: uncoupling protein 1; CON: control; OE, overexpression; HFD: high fat diet; iWAT: inguinal white adipose tissue; eWAT: epididymal white adipose tissue; BAT: brown adipose tissue; HOMA‐IR: homeostasis model assessment of insulin resistance; GTT: glucose tolerance test; ITT: insulin tolerance test; hADP: human adiponectin; NS: no significance; ANOVA: one‐way analysis of variance. All data are presented as mean ± *SEM*. Statistical significance was determined by unpaired two‐tailed student's *t*‐test (E–G, K and M–O) and two‐way ANOVA (B‐C, J and L).

To evaluate the effects of the hADP‐*Ucp1* OE plasmid on obesity development and metabolic homeostasis, mice fed a HFD were treated with the hADP‐*Ucp1* OE plasmid (Figure 5A). Treatment with the hADP‐*Ucp1* OE plasmid significantly attenuated the rate of weight gain in obese mice (Figures 5B and S4A and B), without affecting food intake (Figure 5C). Additionally, the hADP‐*Ucp1* OE plasmid‐treated mice exhibited increased body temperature, BAT temperature and energy expenditure (Figure S4C–G), without causing an increase in corticosterone (a stress‐related hormone, Figure S4H). Moreover, the qPCR analysis results showed that after treatment with hADP‐*Ucp1* OE plasmid, the expression of adipose thermogenic genes increased in iWAT and BAT, while the expression of inflammation‐related genes decreased (Figure S4I and J). The weights of iWAT, eWAT, BAT and liver were notably reduced in HFD‐fed mice treated with the hADP‐*Ucp1* OE plasmid compared to controls (Figure 5D and E). Furthermore, the hADP‐*Ucp1* OE plasmid treatment resulted in a decrease in the serum levels of triglycerides (TG) and the hepatic TG levels (Figure 5F and G). Additionally, it diminished the lipid droplet size in iWAT, eWAT, BAT and liver (Figures 5H and S5A–F). To further evaluate the impact of hADP‐*Ucp1* OE plasmid treatment on obesity, body composition analysis was conducted. Following 12 weeks of the hADP‐*Ucp1* OE plasmid treatment, during which mice exhibited a marked reduction in fasting body weight (Figure S5G), the hADP‐*Ucp1* OE plasmid treatment resulted in a significant decrease in fat mass (Figure S5H), while no loss of lean mass was observed (Figure S5I). Moreover, the hADP‐*Ucp1* OE plasmid treatment significantly increased UCP1 protein expression in iWAT, eWAT, and BAT of HFD‐fed mice (Figures 5H and S6A–F), indicating enhanced thermogenic activity in these adipose tissues. In addition, we found that after hADP‐*Ucp1* OE plasmid treatment, BAT presented multiple thermogenesis adipose features such as increased mitochondrial quantity and mitochondrial crest density (Figure 5I).

We further assessed the metabolic impact of hADP‐*Ucp1* OE plasmid treatment. The results of GTT revealed that treatment with the hADP‐*Ucp1* OE plasmid improved glucose homeostasis in HFD‐fed mice (Figure 5J and K). The results of ITT demonstrated that hADP‐*Ucp1* OE plasmid treatment alleviated insulin resistance in HFD‐fed mice (Figure 5L and M). Additionally, fasting serum insulin levels were reduced following hADP‐*Ucp1* OE plasmid treatment (Figure 5N), and the homeostasis model assessment of insulin resistance (HOMA‐IR) index (a widely used mathematical index to estimate insulin resistance (IR)) showed a significant improvement in hADP‐*Ucp1* OE plasmid‐treated mice (Figure 5O). Collectively, these results indicate that treatment with the hADP‐*Ucp1* OE plasmid induces adipose thermogenesis, combats obesity and improves metabolic homeostasis in obese mice.

### 3.6 The hADP promoter‐modified plasmid achieves selective overexpression of UCP1 protein and thermogenesis in human adipocytes

**FIGURE 6:**
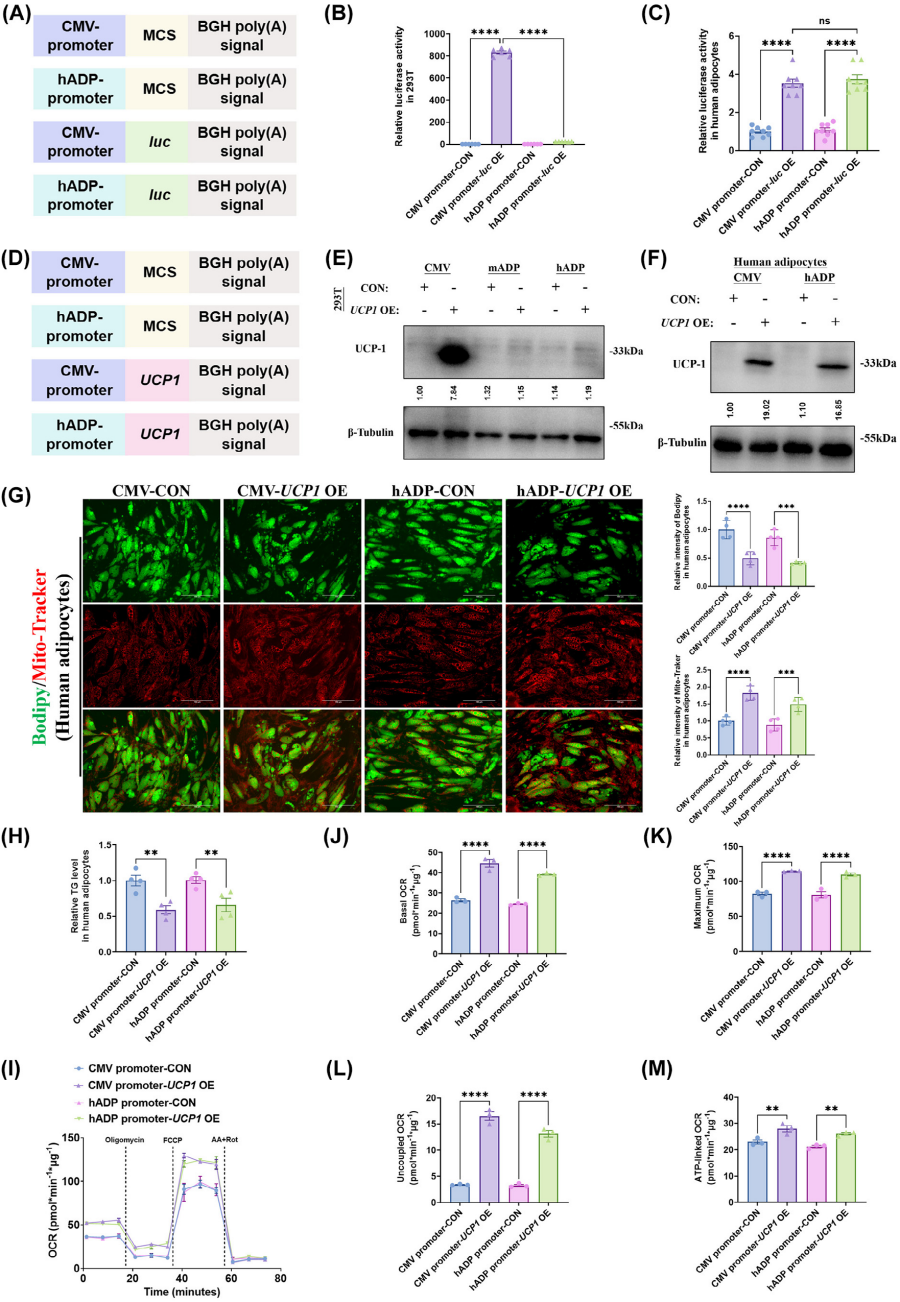
The hADP promoter‐modified plasmids selectively overexpresses UCP1 protein and induces thermogenesis in human adipocytes. (A, D) Schematic diagram of gene elements of different plasmids. (B) Relative luciferase activity analysis in 293T cells transfected with different plasmids. (C) Relative luciferase activity analysis in human mature adipocytes transfected with different plasmids. (E, F) Western blot analysis for UCP1 protein level in 293T cells (E) and human mature adipocytes (F) transfected with different plasmids. The ImageJ software was used for grey scanning. (G) BODIPY green staining for lipid droplet and Mito‐Tracker red staining for mitochondria in human mature adipocytes and staining intensity analysis diagram. Scale bars, 150 µm. (H) The level of intracellular triglyceride in human mature adipocytes. (I–M) OCR (I) and quantification of respiratory profile (J–M) of human mature adipocytes. UCP1: uncoupling protein 1; luc: luciferase; CON: control; OE, overexpression; TG: triglyceride; OCR: oxygen consumption rate; 2‐NBDG: 2‐deoxy‐D‐glucose; CMV: cytomegalovirus; hADP: human adiponectin; NS: no significance; ANOVA: one‐way analysis of variance. All data are presented as mean ± *SEM*. Statistical significance was determined by one‐way ANOVA (B–C, G–H and J–M).

Even though the amino acid sequence of UCP1 is highly conserved between humans and mice (Figure S7A), to assess the translational relevance of our murine studies regarding the hADP promoter‐modified plasmid to human cells, we utilised hADSCs and 293T cells. The effects of the hADP promoter‐modified plasmid on these human cell types were evaluated using luciferase activity assays and western blot analysis (Figure 6A–F). The results of luciferase activity and western blot analysis demonstrated that the hADP promoter‐modified plasmids could specifically overexpress the target protein in human adipocytes derived from hADSCs, but not in non‐adipocytes such as 293T cells and undifferentiated hADSCs (Figures 6A–F and S7B). Furthermore, transfection of human adipocytes with the hADP promoter‐modified *UCP1* overexpression (hADP‐*UCP1* OE) plasmid led to a significant decrease in lipid droplet accumulation and intracellular TG levels (Figure 6G and H), while concurrently enhancing glucose uptake capacity (Figure S7C). Additionally, treatment with the hADP‐*UCP1* OE plasmid markedly increased the basal, maximal, uncoupled, and ATP‐linked OCR in human adipocytes (Figure 6I–M).

### 3.7 Biocompatibility analysis of the hADP‐*Ucp1* OE plasmid treatment

**FIGURE 7:**
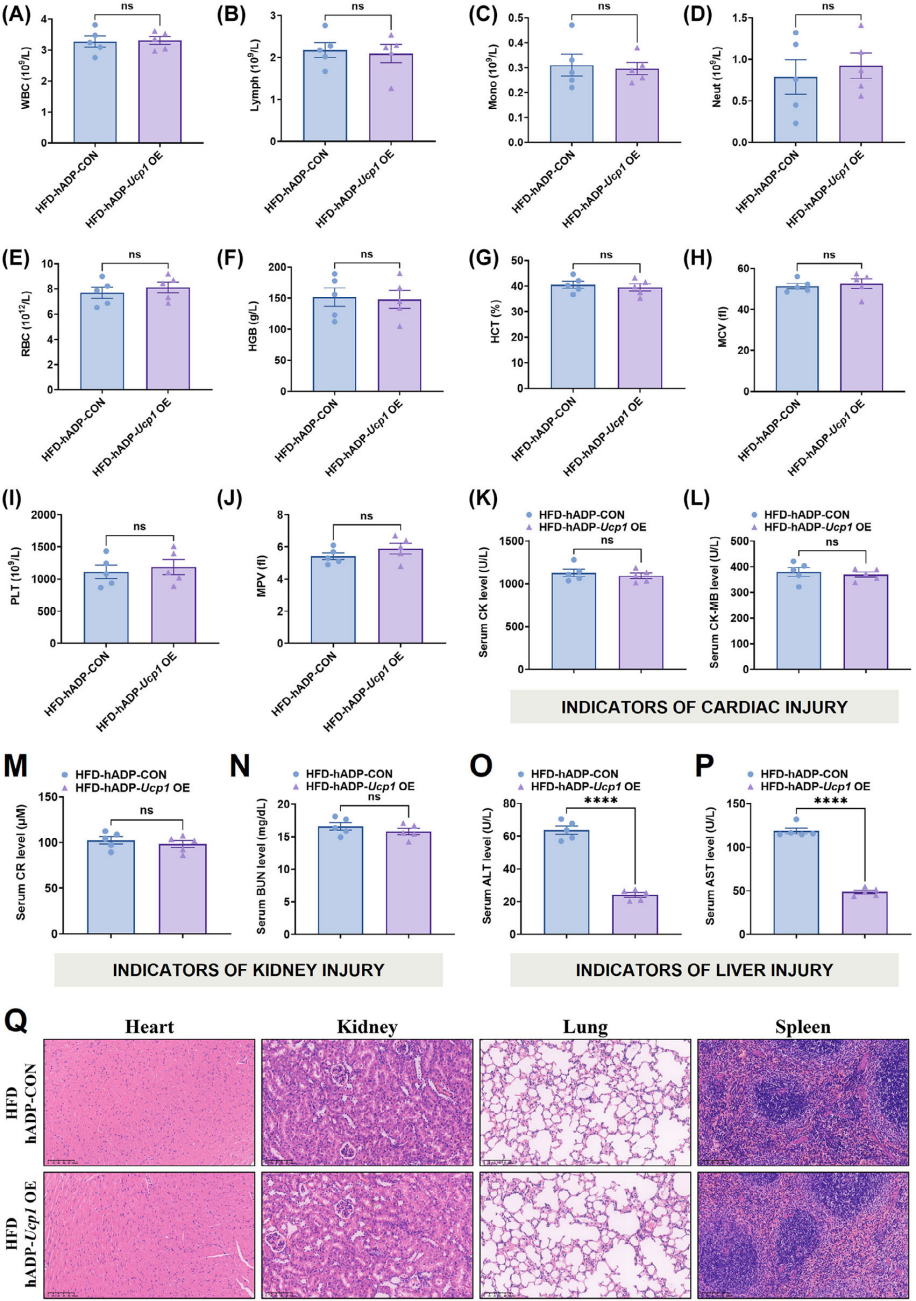
Biocompatibility analysis of the hADP‐*Ucp1* OE plasmid treatment. (A–J) CBC examination in mice treated differently. (K–L) Serum level of CK (K) and CK‐MB (L), the indicators of cardiac injury. (M, N) Serum level of CR (M) and BUN (N), the indicators of kidney injury. (O–P) Serum level of ALT (O) and AST (P), the indicators of liver injury. (Q) Representative images of heart, kidney, lung and spleen stained with haematoxylin and eosin. Scale bars, 100 µm. UCP1: uncoupling protein 1; CON: control; OE, overexpression; hADP: human adiponectin; WBC: white blood cells; Lymph: lymphocytes; Mono: monocytes; Neut: neutrophils; RBC: red blood cells; HGB: haemoglobin; HCT: haematocrit; MCV: mean corpuscular volume; PLT: platelet; MPV: mean platelet volume; CK: creatine kinase; CK‐MB: creatine kinase‐MB; CR: creatinine; BUN: blood urea nitrogen; ALT: alanine aminotransferase; AST: aspartate aminotransferase; NS: no significance. All data are presented as mean ± *SEM*. Statistical significance was determined by unpaired two‐tailed student's *t*‐test (A–P).

During the treatment period, the food intake of the mice treated with hADP‐*Ucp1* OE plasmid did not change (Figures 4C and 5C), indicating that the hADP‐*Ucp1* OE plasmid treatment did not affect the appetite of mice. We further conducted a complete blood count (CBC) examination and examined the indicators of cardiac, hepatic and renal functions after the treatment to evaluate the safety of the hADP‐*Ucp1* OE plasmid treatment. The results showed that the hADP‐*Ucp1* OE plasmid treatment did not cause any significant changes in the level of white blood cells (WBC), lymphocytes (Lymph), monocytes (Mono), neutrophils (Neut), red blood cells (RBC), haemoglobin (HGB), haematocrit (HCT), mean corpuscular volume (MCV), platelet (PLT) and mean platelet volume (MPV) (the results of CBC, Figure 7A–J), the serum level of creatine kinase (CK) and creatine kinase‐MB (CK‐MB) (the indicators of cardiac injury, Figure 7K and L) and the serum level of creatinine (CR) and blood urea nitrogen (BUN) (the indicators of kidney injury, Figure 7M and N). However, the indicators of liver injury, alanine aminotransferase (ALT) and aspartate aminotransferase (AST), significantly decreased in hADP‐*Ucp1* OE plasmid‐treated mice (Figure 7O and P), suggesting that the hADP‐*Ucp1* OE plasmid treatment improved the liver injury caused by HFD‐induced hepatic steatosis. In addition, we evaluated the potential tissue toxicity of the hADP‐*Ucp1* OE plasmid treatment on organs including the heart, kidney, lung and spleen. Haematoxylin and eosin (HE) staining of these organs showed no obvious pathological abnormalities (Figure 7Q), indicating that the hADP‐*Ucp1* OE plasmid treatment exhibited great biocompatibility. These findings suggest that the hADP‐*Ucp1* OE plasmid treatment is safe and effective in managing obesity and metabolic syndrome.

## 4 DISCUSSION

The escalating global obesity crisis underscores the pressing demand for novel therapeutic approaches beyond traditional lifestyle, pharmacological and surgical interventions.^25^, ^28^, ^29^ This study introduces an innovative strategy to obesity management by leveraging an hADP promoter‐modified plasmid to induce adipose‐specific overexpression of UCP1. This strategy selectively enhances adipose thermogenesis and energy expenditure, thereby targeting a distinct pathway from those involved in appetite suppression or surgical intervention.^14^, ^16^, ^18^, ^24^, ^25^, ^28^, ^31^ Our findings demonstrate the efficacy of this plasmid‐based therapy in both murine models and human adipocytes, highlighting its potential to address obesity and its associated metabolic complications. Specifically, the observed reductions in adipose tissue weight, improvements in glucose homeostasis, and increases in energy expenditure in diet‐induced obese mice suggest that hADP promoter‐modified UCP1 overexpression plasmid therapy may have broad applicability in mitigating obesity phenotypes, including those with concomitant metabolic dysfunction. Moreover, the successful validation of this strategy in human adipocytes underscores its translational potential in clinical obesity management (Figure 6). Our research offers a theoretical foundation for subsequent clinical exploration and offers a complementary or alternative solution for patients who have shown limited response to existing therapies.^17^, ^18^, ^19^, ^21^, ^22^, ^27^

Inducing adipose thermogenesis has emerged as a weight loss therapy over the last twenty years.^32^, ^39^, ^54^, ^55^, ^56^, ^57^, ^58^, ^59^, ^60^ The β3‐adrenergic receptor (β3‐AR), a potent target for inducing adipose tissue thermogenesis identified in rodents previously, was found to have poor efficacy in humans during clinical trials, which is attributed to the low expression of β3AR in human adipocytes.^43^, ^61^ On the other hand, the β2‐adrenergic receptor (β2‐AR), which has been identified to exhibit high expression in human adipose tissue, has its further application restricted due to potential cardiovascular side effects.^43^, ^62^ Recently, an increasing number of approaches have been developed to selectively induce adipose thermogenesis, such as modifying delivery vectors, utilising adeno‐associated viruses (AAV) and transplanting CRISPR‐engineered adipocytes.^63^, ^64^, ^65^, ^66^, ^67^, ^68^, ^69^ However, currently, modified delivery vectors mostly achieve adipose‐specific delivery through their physical and chemical properties, which still carries a certain likelihood of off‐target effects. Among gene therapies attracting increasing attention in clinical practice, AAV therapy is limited in its application for non‐genetic diseases due to the high costs associated with its production and transportation.^65^, ^66^, ^67^ An emerging alternative, the CRISPR activation (CRISPRa) system, has shown promising therapeutic potential. Studies have demonstrated that transplanting CRISPR‐engineered, high‐expression UCP1 adipocytes can decrease body weight and improve metabolic homeostasis in obese mice; however, its cost and operational complexity also limit widespread clinical application.^68^, ^69^ In contrast, naked plasmid therapy, a gene therapy that has been developed in recent years, has a much lower cost compared to gene therapies based on viral vectors.^65^, ^66^, ^67^, ^70^ It has been used to treat chronic non‐genetic diseases such as lower limb ischemic diseases, and clinical trials have shown its effectiveness and safety.^46^, ^47^, ^48^ However, to date, there is still no plasmid therapy that can achieve adipose‐specific overexpression by modifying plasmids.

This study investigated the feasibility of inducing adipose‐selective UCP1 overexpression via modified plasmids as a novel therapeutic strategy for obesity. Our results demonstrated that the hADP promoter‐modified plasmid achieved selective overexpression of UCP1 protein in mouse adipocytes and adipose tissue. The hADP promoter‐modified plasmid drove robust overexpression of the UCP1‐6×HIS protein in iWAT, eWAT and BAT, while no detectable expression was observed in non‐adipose tissues (Figure 2). This selective overexpression is crucial for minimising off‐target effects and guaranteeing the safety and effectiveness of the therapy. In vivo experiments further demonstrated the efficacy of the hADP‐*Ucp1* OE plasmid in promoting thermogenesis and energy expenditure (Figures 4 and S4). We believe that the enhanced metabolic rate caused by increased oxygen consumption and UCP1 overexpression is probably a key factor contributing to fat loss. UCP1, as a key protein regulating thermogenesis in BAT, can increase energy dissipation in the form of heat and elevating overall energy expenditure.^41^, ^42^ This enhanced energy metabolism may directly promote the breakdown of fat stores to meet the increased energy demand, which is consistent with the observed fat loss. However, we do not rule out the involvement of other pathways. For example, UCP1 overexpression may affect lipid metabolism‐related signalling pathways, such as regulating the genes related to lipolysis or fatty acid oxidation, thereby accelerating fat decomposition.^33^, ^41^, ^71^ Furthermore, UCP1 upregulation followed by adipose implantation was recently shown to be beneficial as a cancer therapy.^72^ Due to the similar mechanism, hADP‐*Ucp1* OE therapy may have similar effects and deserves further research. The hADP‐*Ucp1* OE plasmid also showed promising effects in weight loss and improving glucose metabolism (Figure 5). These findings suggest that the hADP‐*Ucp1* OE plasmid‐based therapy induces adipose thermogenesis in obese mice, thereby mitigating obesity and glucose intolerance.

The translational relevance of our murine studies was further supported by the results in human adipocytes. The hADP promoter‐modified plasmid could specifically overexpress the target protein in human adipocytes derived from hADSCs, but not in non‐adipocytes such as 293T cells and undifferentiated hADSCs (Figures 6 and S7). Transfection of human adipocytes with the hADP‐*UCP1* OE plasmid led to a marked decrease in lipid droplet accumulation and intracellular TG levels, while enhancing glucose uptake capacity. Additionally, the hADP‐*UCP1* OE plasmid markedly enhanced thermogenic activity in human adipocytes. The decrease in ALT and AST levels (Figure 7O and P) and improvement in liver histology (Figure S5F) indicate that hADP‐*Ucp1* OE treatment can significantly improve obesity induced metabolic dysfunction associated fatty liver disease (MASLD) in mice. In addition, the biocompatibility analysis results showed that hADP‐*Ucp1* OE plasmid therapy did not cause widespread inflammation (Figure 7A–D) and was overall safe (Figure 7), highlighting the advantages of plasmid therapy over viral gene therapy. These findings suggest that the hADP promoter‐modified plasmid‐based therapies is a promising therapeutic approach for obesity and MASLD in humans.

Although the above results are promising, several limitations restrict the clinical translation of our findings. Firstly, although this therapy has demonstrated preclinical efficacy in male mouse models. These models cannot fully reflect the pathophysiology of human obesity, particularly since only male mice were used. Given known sex differences in adipose tissue biology and metabolism, future studies should include female models and, ideally, primates to comprehensively evaluate efficacy, safety, and the translational potential of this therapy. Secondly, the delivery efficiency of plasmid‐based therapies in humans remains a challenge,^66^, ^70^ due to obstacles in tissue‐specific uptake and sustained expression of plasmids. Optimising delivery vehicles to enhance adipose targeting and minimise systemic exposure is crucial. Thirdly, although the non‐integration of plasmids into the genome underscores their safety profile, this also leads to shorter sustained efficacy of plasmid drugs compared to other gene therapies,^68^, ^69^, ^70^ requiring more frequent injections (such as twice a week in this study). Additionally, while this study focused on UCP1 overexpression driven by the hADP promoter‐modified plasmid, future research should explore optimisation of promoters or gene codons to further enhance tissue specificity and expression efficiency. Finally, although the weight loss and metabolic improvement effects (such as glucose tolerance and insulin sensitivity) of this therapy have been observed to be effective, combination therapy or adjuvant interventions may still be necessary to achieve clinically meaningful outcomes.

In conclusion, our study demonstrates that the hADP promoter‐modified plasmid achieves adipose‐selective overexpression of UCP1 protein and induces adipose thermogenesis, leading to reduced weight gain, improved metabolic homeostasis, and enhanced energy expenditure in mice. The translational potential of this approach is further supported by the results in human adipocytes. Subsequent investigations need to centre on optimising the delivery system, assessing the safety and effectiveness of the therapy, and exploring their potential clinical applications in humans. The development of this novel therapeutic strategy offers a promising and innovative strategy for the clinical management of obesity.

## AUTHOR CONTRIBUTIONS

**Ze‐Wei Zhao**: Investigation; conceptualisation; visualisation; methodology; data curation; formal analysis; writing–original draft; writing–review and editing. **Longyun Hu**: Data curation; investigation; formal analysis. **Bigui Song**: Data curation; investigation. **Qian Wu**: Data curation; formal analysis. **Jiejing Lin**: Data curation; formal analysis. **Qingqing Liu**: Data curation; formal analysis. **Siqi Liu**: Data curation; formal analysis. **Jin Li**: Data curation; formal analysis. **Molin Wang**: Data curation; formal analysis. **Jin Li**: Funding acquisition; supervision; project administration; writing–review and editing. **Zhonghan Yang**: Funding acquisition; conceptualisation; project administration; supervision; writing–review and editing.

## CONFLICT OF INTEREST STATEMENT

The authors have declared that no competing interests exist.

## ETHICS STATEMENT

All the animal experiments were conducted with the approval of the Animal Care and Use Committee of Sun Yat‐sen University (Approval No. SYSU‐IACUC‐2024‐000465). This study was conducted in accordance with the ethical principles derived from the Declaration of Helsinki and Belmont Report and was approved by the review board of Sun Yat‐sen University (Guangzhou, China).

## Supporting information



Supporting Information

## Data Availability

The data that support the findings of this study are available from the corresponding author upon reasonable request. The RPKM data of *ADIPOQ* gene in human tissues can be obtained from HPA RNA‐seq normal tissues (PRJEB4337, https://www.proteinatlas.org/ENSG00000181092‐ADIPOQ/tissue).^74^ The nTPM data of ADIPOQ gene in human tissues can be obtained from GTEx Portal database (https://gtexportal.org/home/multiGeneQueryPage).^75^
