# Supplementary material for: DNA‐mediated UCP1 overexpression in adipose tissue: A promising anti‐obesity gene therapy
Source: Clin Transl Med. 2025 Oct 2;15(10):e70491. doi: 10.1002/ctm2.70491 (PMC12491148; doi:10.1002/ctm2.70491)
Supplement: Supplementary file 1 — Supporting Information [file CTM2-15-e70491-s001.docx]

**Supplemental figures and figure legends**


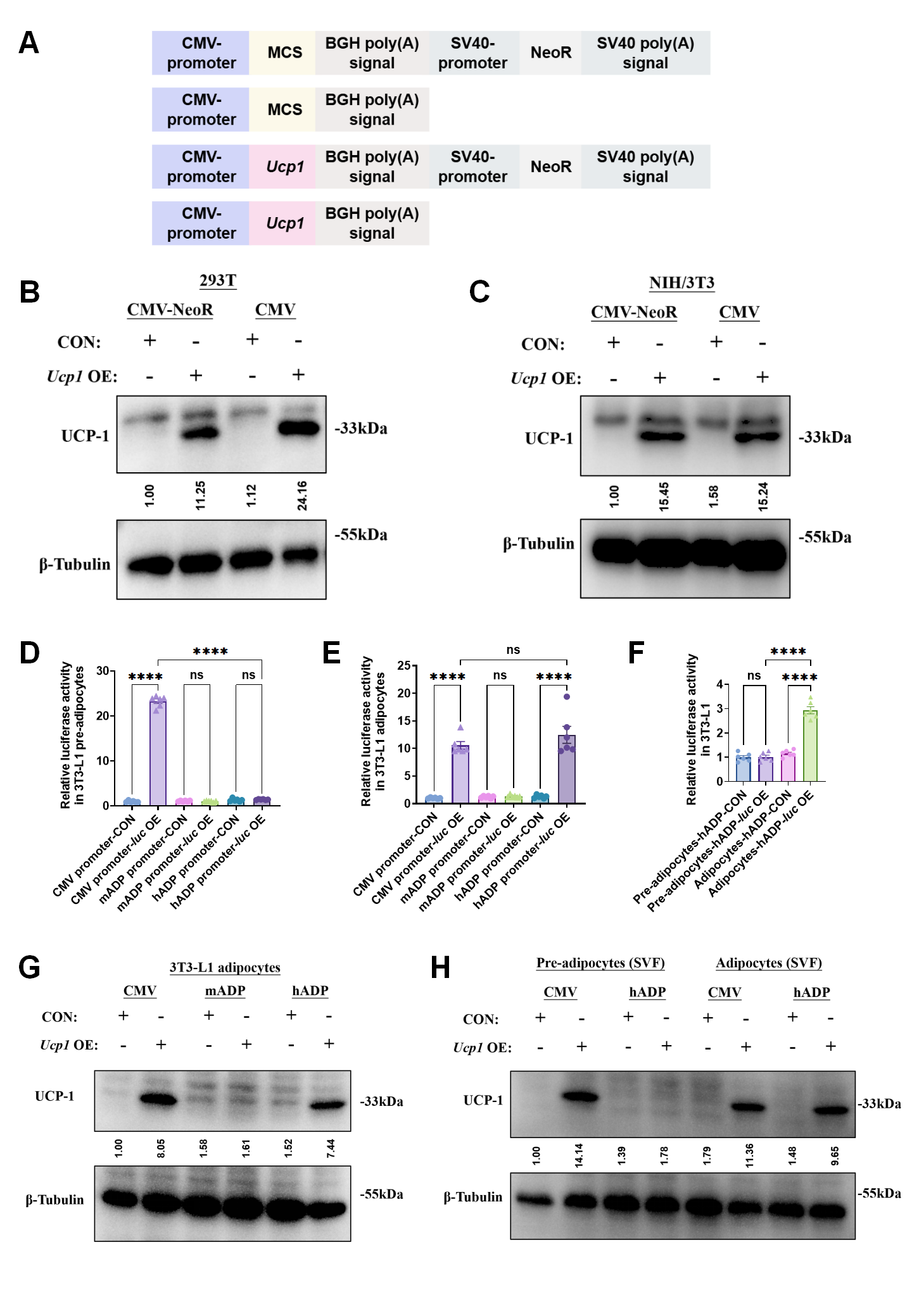


**Figure S1**. **The hADP promoter-modified plasmids achieves selective overexpression of target protein in mouse adipocytes, in contrast to the mADP promoter.** (**A**) Schematic diagram of gene elements of different plasmids. (**B-C**) Western blot analysis for UCP1 protein level in 293T cells (**B**) and NIH/3T3 cells (**C**) transfected with different plasmids. The ImageJ software was used for gray scanning. (**D**) Relative luciferase activity analysis in 3T3-L1 pre-adipocytes transfected with different plasmids. (**E**) Relative luciferase activity analysis in 3T3-L1 mature adipocytes transfected with different plasmids. (**F**) Relative luciferase activity analysis in 3T3-L1 pre-adipocytes and mature adipocytes transfected with different plasmids. (**G**) Western blot analysis for UCP1 protein level in 3T3-L1 mature adipocytes transfected with different plasmids. (**H**) Western blot analysis for UCP1 protein level in mouse primary pre-adipocytes and adipocytes transfected with different plasmids. The ImageJ software was used for gray scanning. UCP1: uncoupling protein 1; luc: luciferase; CON: control; OE: overexpression; SV40: simian virus 40; NeoR: neomycin resistance gene; CMV: cytomegalovirus; mADP: mouse adiponectin; hADP: human adiponectin; NS: No Significance; ANOVA: one-way analysis of variance. All data are presented as mean ± *SEM*. Statistical significance was determined by one-way ANOVA (**D-F**).


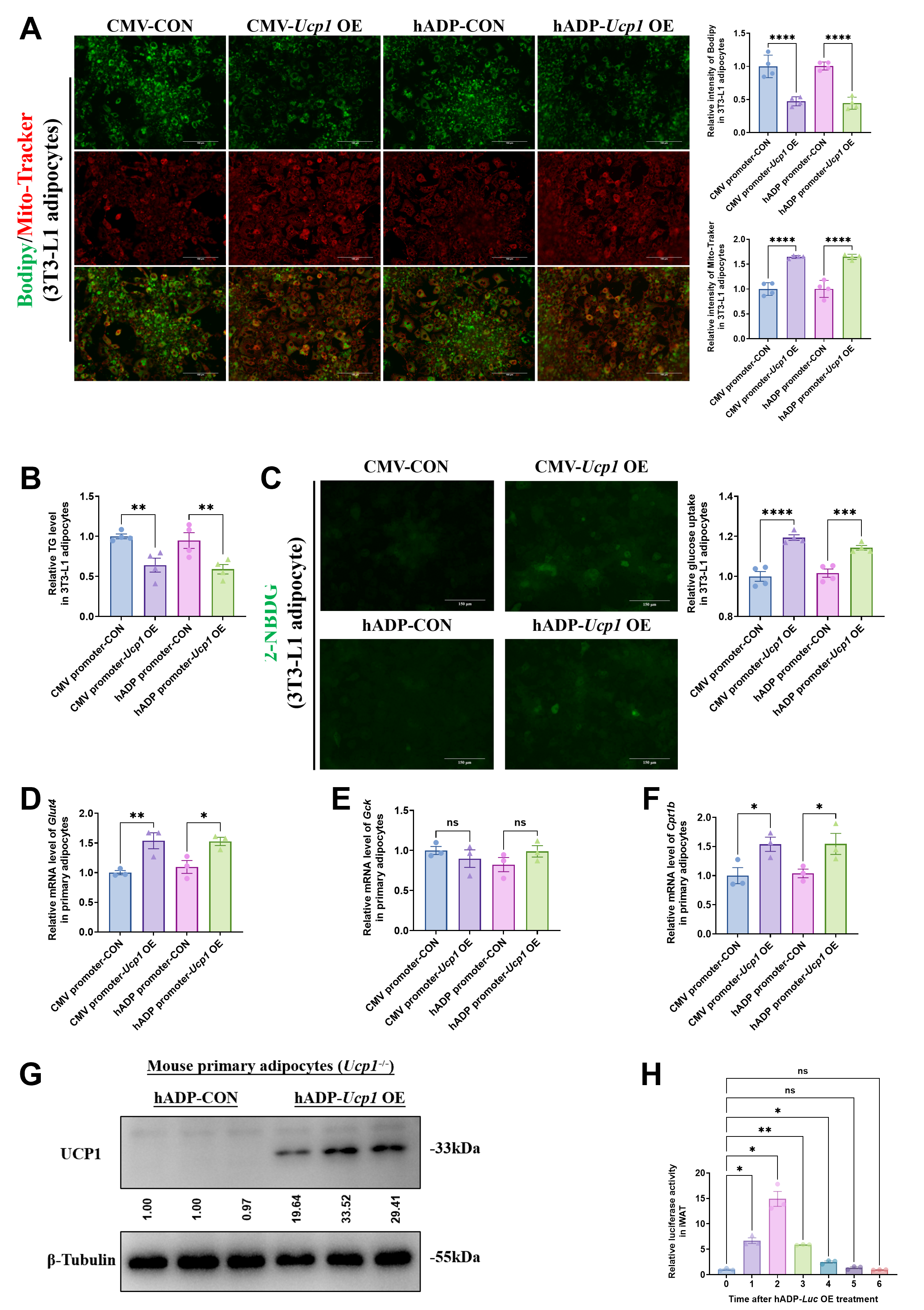


**Figure S2**. **Overexpression of UCP1 driven by the hADP promoter induces lipid droplet reduction in 3T3-L1 adipocytes, consistent with the CMV promoter.** (**A**) Bodipy green staining for lipid droplet and Mito-Tracker red staining for mitochondria in 3T3-L1 mature adipocytes and staining intensity analysis diagram. Scale bars, 150 μm. (**B**) The level of intracellular triglyceride in 3T3-L1 mature adipocytes. (**C**) Glucose uptake assay in 3T3-L1 mature adipocytes and staining intensity analysis diagram. (**D-F**) qPCR analysis for *Glut4* (D), *Gck* (E) and *Cpt1b* (F) mRNA level in mouse primary adipocytes transfected with different plasmids. (**G**) Western blot analysis for UCP1 protein level in mouse primary adipocytes transfected with different plasmids. Primary adipocytes were differentiated from SVF isolated from UCP1 knockout (*Ucp1*^-/-^) mice. The ImageJ software was used for gray scanning. (**H**) Relative luciferase activity analysis in iWAT treated differently. UCP1: uncoupling protein 1; CON: control; OE, overexpression; TG: triglyceride; 2-NBDG: 2-deoxy-D-glucose; *Ucp1*^-/-^: UCP1 knockout; CMV: cytomegalovirus; hADP: human adiponectin; SVF: stromal vascular fraction; NS: No Significance; ANOVA: one-way analysis of variance. All data are presented as mean ± *SEM*. Statistical significance was determined by one-way ANOVA (**A-F and H**).


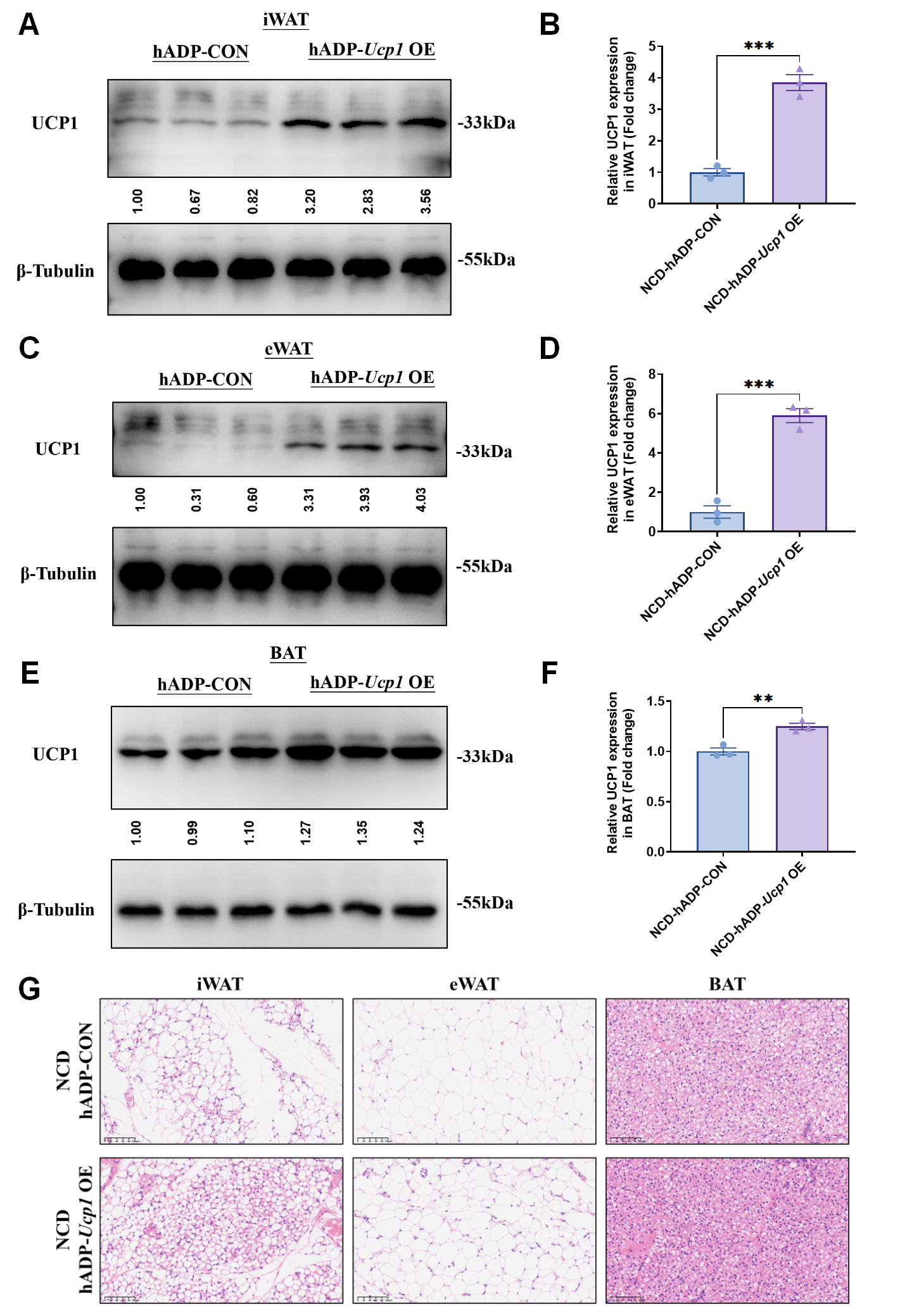


**Figure S3**. **Characterization of NCD-fed mice after hADP-*Ucp1* OE treatment.** (**A-F**) Western-blot analysis for the level of UCP1 protein in iWAT (**A-B**), eWAT (**C-D**) and BAT (**E-F**) from differently treated mice. The ImageJ software was used for gray scanning. (**G**) Representative images of iWAT, eWAT and BAT stained with hematoxylin and eosin. Scale bars, 100 μm. UCP1: uncoupling protein 1; CON: control; OE, overexpression; NCD: normal-chow diet; iWAT: inguinal white adipose tissue; eWAT: epididymal white adipose tissue; BAT: brown adipose tissue; hADP: human adiponectin. All data are presented as mean ± *SEM*. Statistical significance was determined by unpaired two-tailed Student’s t-test (**B, D and F**).


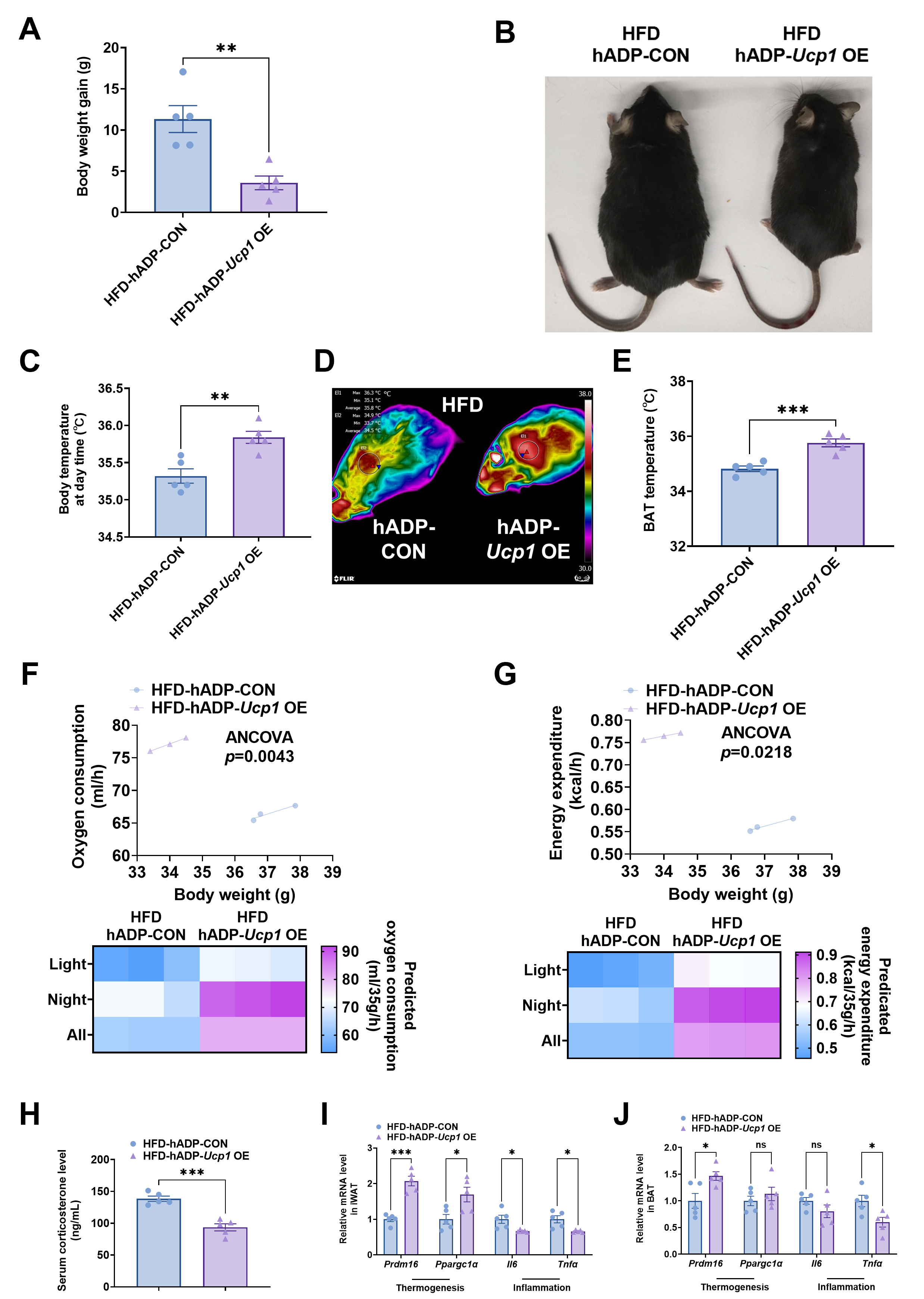


**Figure S4**. **The hADP-*Ucp1* OE treatment promotes thermogenesis and energy expenditure in mice fed with a HFD.** (**A**) Body weight gain of mice treated differently. (**B**) Representative image of mice treated differently. (**C**) Body temperature of mice treated differently. (**D-E**) Representative thermal image and BAT temperature (**E**) of mice treated differently. (**F-G**) The oxygen consumption (**F**) and the calorie consumption (**G**) of C57BL/6J mice fed with a HFD for 12 weeks (n = 3 per treatment). Data in panels F and G have been analyzed using ANCOVA with oxygen consumption (F)/ calorie consumption (G) as dependent variable, group as fixed variable and body mass as covariate. (**H**) The serum corticosterone level in differently treated mice. (**I-J**) qPCR analysis for *Prdm16*, *Ppargc1**α*, *Il6* and *Tnfα* mRNA level in iWAT (**I**) and BAT (**J**). UCP1: uncoupling protein 1; CON: control; OE, overexpression; HFD: high fat diet; iWAT: inguinal white adipose tissue; BAT: brown adipose tissue; hADP: human adiponectin. All data are presented as mean ± *SEM*. Statistical significance was determined by unpaired two-tailed Student’s t-test (**A, C, E and H-J**). Metabolic cage results were analyzed by performing ANCOVA (**F-G**).


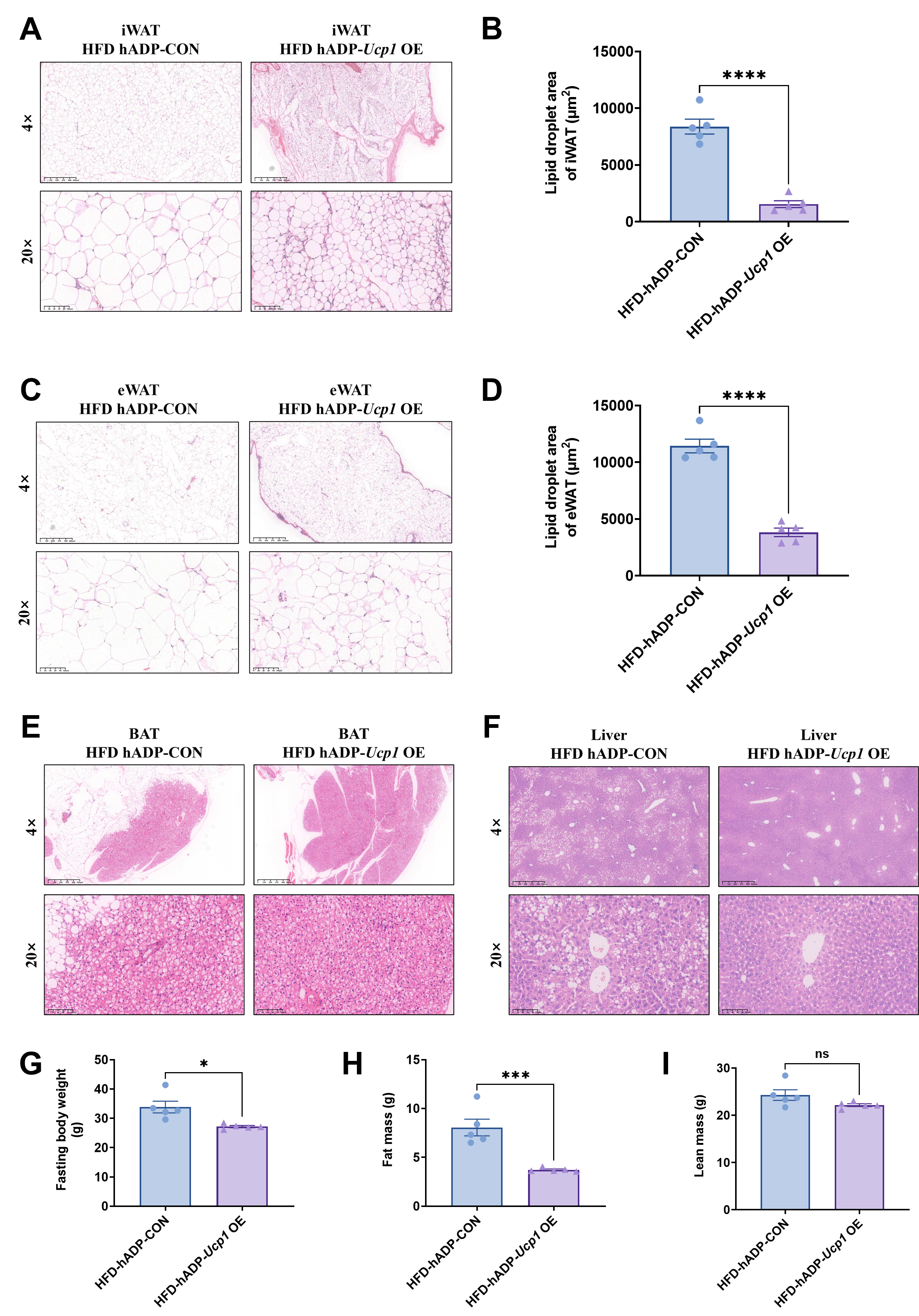


**Figure S5**. **Characterization of HFD-fed mice after hADP-*Ucp1* OE treatment.** (**A, C and E-F**) Representative images of iWAT (**A**), eWAT (**C**), BAT (**E**) and liver (**F**) stained with hematoxylin and eosin. 4×: Scale bars, 620 μm; 20×: Scale bars, 100 μm. (**B and D**) Area of lipid droplets in iWAT (**B**) and eWAT (**D**). (**G-I**) Fasting body weight (**G**), fat mass (**H**) and lean mass (**I**) in C57BL/6J DIO mice after different treatment. UCP1: uncoupling protein 1; CON: control; OE, overexpression; HFD: high fat diet; iWAT: inguinal white adipose tissue; eWAT: epididymal white adipose tissue; BAT: brown adipose tissue; NS: No Significance. All data are presented as mean ± *SEM*. Statistical significance was determined by unpaired two-tailed Student’s t-test (**B, D and G-I**).

**
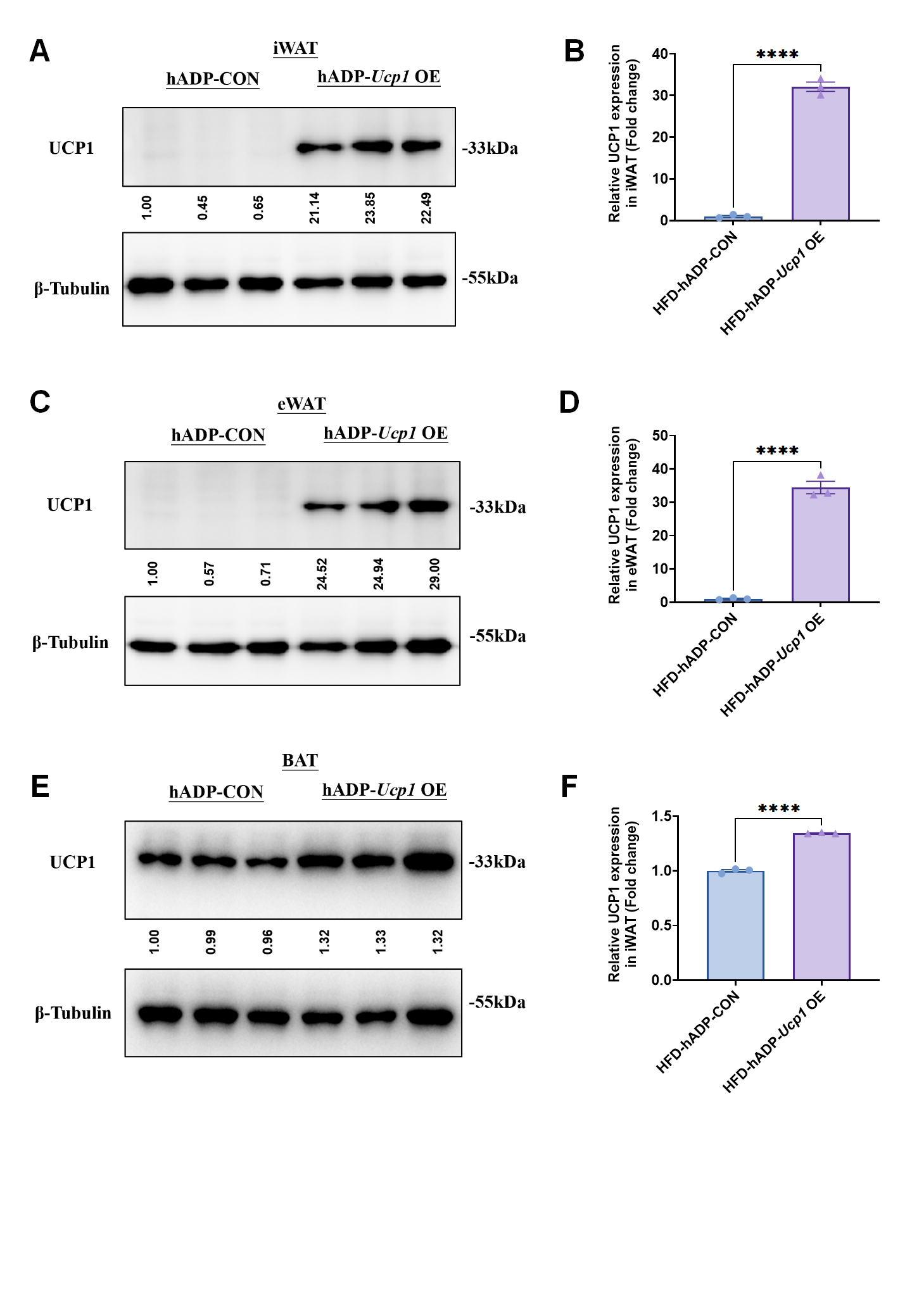
Figure S6**. **The hADP-*Ucp1* OE treatment induce browning of adipose tissue in HFD-fed mice.** (**A-F**) Western-blot analysis for the level of UCP1 protein in iWAT (**A-B**), eWAT (**C-D**) and BAT (**E-F**) from differently treated mice. The ImageJ software was used for gray scanning. UCP1: uncoupling protein 1; CON: control; OE, overexpression; HFD: high fat diet; iWAT: inguinal white adipose tissue; eWAT: epididymal white adipose tissue; BAT: brown adipose tissue. All data are presented as mean ± *SEM*. Statistical significance was determined by unpaired two-tailed Student’s t-test (**B, D and F**).


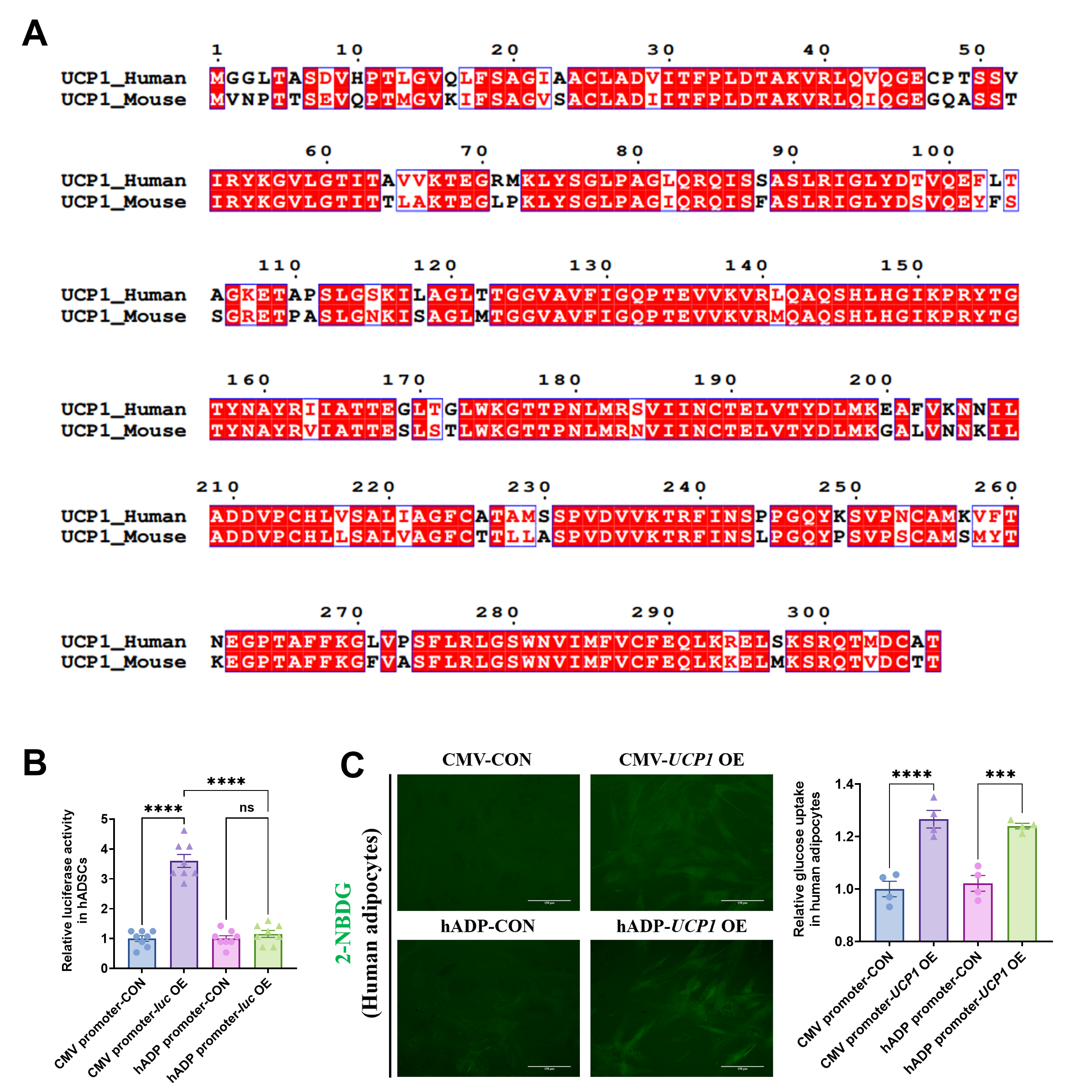
**Figure S7**. **Overexpression of UCP1 driven by hADP promoter enhances glucose uptake in human adipocytes.** (**A**) Amino acid sequence alignment map of UCP1 in human and mouse. (**B**) Relative luciferase activity analysis in undifferentiated hADSCs transfected with different plasmids. (**C**) Glucose uptake assay in human mature adipocytes and staining intensity analysis diagram. UCP1: uncoupling protein 1; luc: luciferase; CON: control; OE, overexpression; 2-NBDG: 2-deoxy-D-glucose; CMV: cytomegalovirus; hADP: human adiponectin; NS: No Significance; ANOVA: one-way analysis of variance. All data are presented as mean ± *SEM*. Statistical significance was determined by one-way ANOVA (**B-C**).
